# Harvesting interacts with climate change to affect future habitat quality of a focal species in eastern Canada’s boreal forest

**DOI:** 10.1371/journal.pone.0191645

**Published:** 2018-02-07

**Authors:** Junior A. Tremblay, Yan Boulanger, Dominic Cyr, Anthony R. Taylor, David T. Price, Martin-Hugues St-Laurent

**Affiliations:** 1 Sciences and Technology Branch, Environment and Climate Change Canada, Québec, Québec, Canada; 2 Natural Resources Canada, Canadian Forest Service, Laurentian Forestry Centre, Stn. Sainte-Foy, Québec, Québec, Canada; 3 Natural Resources Canada, Canadian Forest Service, Atlantic Forestry Centre, Fredericton, New Brunswick, Canada; 4 Natural Resources Canada, Canadian Forest Service, Northern Forestry Centre, Edmonton, Alberta, Canada; 5 Université du Québec à Rimouski, Centre for Northern Studies & Centre for Forest Research, Département de biologie, chimie et géographie, Allée des Ursulines, Rimouski, Québec, Canada; University of the Chinese Academy of Sciences, CHINA

## Abstract

Many studies project future bird ranges by relying on correlative species distribution models. Such models do not usually represent important processes explicitly related to climate change and harvesting, which limits their potential for predicting and understanding the future of boreal bird assemblages at the landscape scale. In this study, we attempted to assess the cumulative and specific impacts of both harvesting and climate-induced changes on wildfires and stand-level processes (e.g., reproduction, growth) in the boreal forest of eastern Canada. The projected changes in these landscape- and stand-scale processes (referred to as “drivers of change”) were then assessed for their impacts on future habitats and potential productivity of black-backed woodpecker (BBWO; *Picoides arcticus*), a focal species representative of deadwood and old-growth biodiversity in eastern Canada. Forest attributes were simulated using a forest landscape model, LANDIS-II, and were used to infer future landscape suitability to BBWO under three anthropogenic climate forcing scenarios (RCP 2.6, RCP 4.5 and RCP 8.5), compared to the historical baseline. We found climate change is likely to be detrimental for BBWO, with up to 92% decline in potential productivity under the worst-case climate forcing scenario (RCP 8.5). However, large declines were also projected under baseline climate, underlining the importance of harvest in determining future BBWO productivity. Present-day harvesting practices were the single most important cause of declining areas of old-growth coniferous forest, and hence appeared as the single most important driver of future BBWO productivity, regardless of the climate scenario. Climate-induced increases in fire activity would further promote young, deciduous stands at the expense of old-growth coniferous stands. This suggests that the biodiversity associated with deadwood and old-growth boreal forests may be greatly altered by the cumulative impacts of natural and anthropogenic disturbances under a changing climate. Management adaptations, including reduced harvesting levels and strategies to promote coniferous species content, may help mitigate these cumulative impacts.

## Introduction

Climate change is expected to have a strong impact on global biodiversity [1], including bird species [2–4]. Recent northward range expansions of breeding birds have already been documented and attributed to climate change in temperate North America [5], as well as in Europe [4,6]. Climate change-driven range shifts are projected to be most dramatic at northern latitudes, where polar amplification is driving more rapid warming than the global mean [7]. As such, species currently restricted to boreal regions may experience range decrease due to the reduction in areas of suitable biomes caused by northward shifts in climate zones, such as projected for North America [8]. However, positive temperature affinities and broad climatic tolerance suggest that many other species may expand their breeding distributions within the boreal region [9].

Many boreal bird species rely on specific vegetation characteristics for different life history traits (e.g., nesting, feeding) that may be associated with different habitat types. Changes in boreal forest properties are thus likely to greatly affect habitat availability, triggering potential shifts in species demography and population persistence [10], and thus in distribution range [3]. Indeed, climate change within boreal regions has already led to increased drought- and insect-induced tree mortality [11, 12], changes in forest productivity [13], wetland drying [14], and increased wildfire activity [15, 16]. Furthermore, there is evidence of northward shifts in tree species’ ranges in recent decades, notably at the temperate-boreal interface (e.g., [17]). Anthropogenic climate forcing over the coming decades is projected to drive significant changes in boreal forest composition and age structure [18, 19] through changes at the stand (e.g., mortality, competition, reproduction, growth) and landscape levels (e.g., natural disturbances) that are likely to impact the distribution of boreal bird species habitats. Indeed, current studies show that slow climate-induced vegetation changes have strongly constrained the poleward or altitudinal migration of some bird species (e.g., [6, 20]), thus underlining the importance of including vegetation patterns when projecting future bird ranges.

In addition to climate change, several studies have already pointed out that anthropogenic activities such as forestry and the extraction of mineral and energy resources, have a very significant impact on boreal bird assemblages, notably by modifying forest vegetation composition [21–23]. Also, these multiple stressors may interact between themselves to amplify impacts on boreal bird habitats (e.g., [24, 25]). For instance, Ordonez et al. [26] reported that areas exposed to both rapid climate warming and land-use changes are expected to undergo the fastest changes in biodiversity and ecosystem function in the continental United States. Recent climatic changes have already been identified as important drivers of bird population dynamics in Sweden, adding to the effects of other drivers such as land-use changes [27, 28]. Thus, the threat of climate change should be integrated with threats attributable to industrial development. In the eastern boreal region forest, harvesting is the most widespread industrial activity [29]; however, the cumulative impacts of both harvesting and climate change on habitat distribution have yet to be quantified for the majority of boreal bird species.

In addition to assessing cumulative impacts, disentangling and assessing the relative contributions of stand- and landscape-scale drivers of changes in vegetation on bird habitat are essential to understand how climate change and harvesting will influence the future of boreal bird assemblages. Recent analyses [30] have suggested that climate-induced changes in stand-scale processes, as well as changes in the fire regime, were more likely to impact future southern Canadian boreal forest landscapes than harvesting. Although the relative importance of harvesting and climate change impacts varies strongly, both regionally and globally [31, 32], relatively little is known about how this affects bird habitats. Identifying and quantifying the impacts of the key drivers of change should facilitate the adaptation of current forest management strategies to minimize negative consequences [30].

Most studies projecting future ranges of bird species rely on correlative species distribution models based mainly on projections of where climate conditions within the current species ranges are likely to occur in the future (see [33]). This approach does not take into account the projected migration of the habitat (e.g., forest cover) as it fails to consider the interactions among stand- and landscape-level impacts of climate change, tree dispersal abilities, and effects of anthropogenic disturbances on forest structure and hence on habitat distribution. Forest landscape models simulate stand- (e.g., forest succession, growth) and landscape-scale processes (e.g., seed dispersal, natural and anthropogenic disturbances; [34]) at temporal and spatial scales that make it possible to characterize wildlife habitats. Furthermore, forest landscape models can simulate the impact of climate change on these ecological processes [35] and, as such, may be useful to project the impacts of climate change on bird habitats. By doing so, forest landscape models allow more realistic projections of bird habitats than species distribution models by improving the representation of spatial legacies of the landscape, future forest disturbance dynamics, vegetation trajectories, and hence bird habitats in a climate change context [36–37].

The Black-backed Woodpecker (BBWO, *Picoides arcticus*) is found in conifer-dominated over-mature and old-growth forests [38–40], and in forest stands recently disturbed by wildfire or insect outbreak [41, 42]. Territory sizes diminish according to prey abundance (mostly bark and wood-boring insects), which is dependent on the availability of recent deadwood [43] and related to forest age and incidence of disturbance [38, 43–44]. BBWO is considered an indicator species for recent deadwood and old-growth biodiversity in the boreal forest [38, 45–46], and has been identified to be potentially threatened by climate change [2]. Moreover, woodpecker abundance has been related to forest bird species richness [47–48]. Hence, we consider that BBWO represents a valuable focal species to understand the impacts of climate change on the biodiversity associated with recent deadwood and old-growth boreal forests.

In this paper, we used the LANDIS-II forest landscape model to project the cumulative impacts of climate change and harvesting on the productivity of BBWO habitat across a large boreal forest landscape in eastern Canada. We translated forest attributes into BBWO habitats, and simulated BBWO potential productivity (number of fledglings) as a proxy to quantify the magnitude of climate change impacts. Furthermore, we assessed the relative importance of climate-induced changes in forest habitat on BBWO productivity occurring (i) at the stand scale (e.g., changes caused by changes in tree reproduction or growth); (ii) those resulting from changes in the fire regime; and (iii) those related to harvesting.

## Material and methods

### Study area

**Fig 1:**
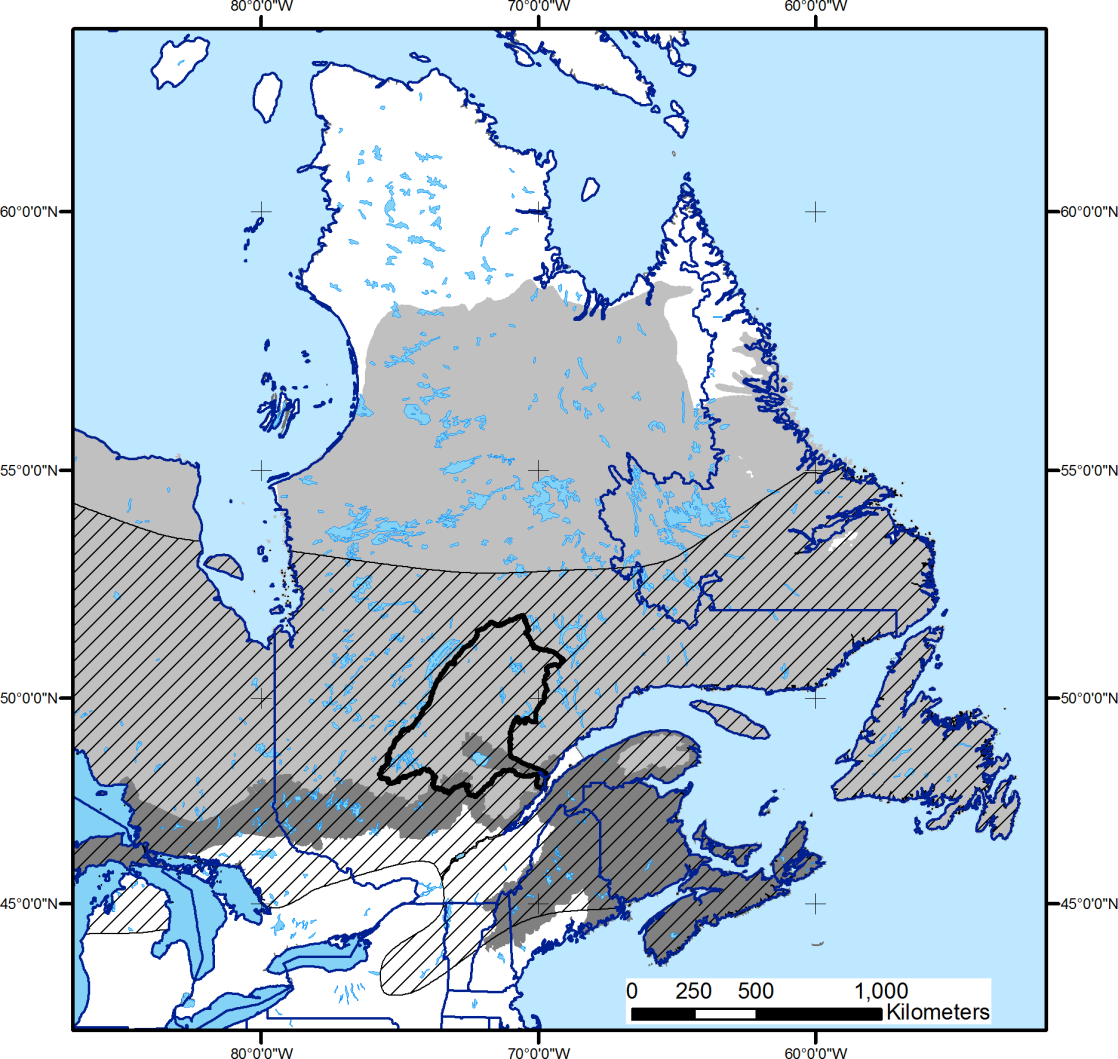
Black-backed Woodpecker range (hatched lines), location of the area where forest landscapes were simulated (delineated in black), and boreal (light gray) and hemiboreal (dark gray) zones (following [49]).

The study region is located in the boreal forest of central Quebec and covers a total of 11.3 Mha (Fig 1). Of this area, about 9.52 Mha are considered productive forest. The remaining unproductive portions are made up of water bodies, wetlands, and barren areas that do not change through the course of our simulations.

Landform and soils in this region are typical of the Canadian boreal shield, dominated by a broadly rolling mosaic of uplands and wetlands where Precambrian granitic bedrock outcrops alternate with ridged to hummocky deposits of coarse-textured mineral soils of glacial origin [50].

The proportion of coniferous tree species, mostly balsam fir (*Abies balsamea*), black spruce (*Picea mariana*), and jack pine (*Pinus banksiana*), increases with latitude, and decreases for boreal deciduous species, such as trembling aspen (*Populus tremuloides*) and white birch (*Betula papyrifera*) [51]. Mesophytic species typical of the mixed and temperate forest, including red (*Acer rubrum*) and sugar (*Acer saccharum*) maples, American beech (*Fagus grandifolia*), red (*Picea rubens*) and white (*Picea glauca*) spruces, and yellow birch (*Betula alleghanensis*), are mostly encountered in the southernmost portions of the study area. Large and relatively frequent stand-replacing fires mostly occur within the northern half of the study area [52, 53] whereas recurrent spruce budworm outbreaks are the most important natural disturbance in the more southern mixed forest portions [54, 55]. Commercial harvesting activities are currently practised in almost all of the study area [56, 57].

### Climate scenarios

Future climate scenarios were built by merging projections of future monthly changes derived from the Canadian Earth System Model version 2 (CanESM2) [58], with 30-yr monthly climate normals for 1961–1990 interpolated from climate station records [59]. Future climate projections from CanESM2 were downloaded from the World Climate Research Program (WCRP) Climate Model Intercomparison Project Phase 5 (CMIP5) archive for each of three different radiative forcing scenarios, known as Representative Concentration Pathways (RCP; e.g., [60]), namely RCP 2.6, RCP 4.5 and RCP 8.5. Monthly time-series of current climate (2000–2010) were interpolated from climate station records using the data of McKenney et al. [59]. According to CanESM2 projections, mean annual temperature would increase by about 3.5°C (RCP 2.6) to 7.5°C (RCP 8.5) throughout the southern boreal region by 2100 (compared with c. 2000), while average precipitation is projected to increase by 10 to 25% (S1 Appendix). Data from CanESM2 for the 1961–2100 period were bias-corrected by expressing them as differences from (temperature) or ratios of (precipitation) CanESM2 monthly means for the 1961–1990 period [59].

### Models

#### LANDIS-II, simulation setup and initial conditions

LANDIS-II is a spatially explicit model that was designed to simulate forest landscapes >10^5^ ha in size [35]. It comprises a library of model extensions to simulate a variety of stand- and landscape-level ecosystem processes, and a core module that manages interactions among the extensions [34]. In LANDIS-II, the forest landscape is represented by a grid of interacting cells within which stand-level forest processes occur, while landscape-level processes, such as tree seed dispersal and disturbances, generally affect multiple cells in a spatially interactive manner. Cell resolution and time steps in LANDIS-II are defined by the user, allowing different time steps to be set for each extension.

In this experiment, grid cell resolution was set to 250 m (6.25 ha) and simulations were run at a 5-yr time step across all activated extensions. All cells were assigned user-defined landtypes that represent relatively homogeneous soil and climate conditions (as described below). Seed dispersal occurs following the algorithm described in Ward et al. [61], which mainly consists of a two-part exponential decay probability distribution with increasing distance.

Forest composition and structure in each grid cell were initialized by combining estimates of the aboveground biomass (AGB) for all tree species based on MODIS imagery [50] obtained from the Canadian National Forest Inventory (NFI; https://nfi.nfis.org) and cohort data from provincial forest inventory plots (FIP). This allowed remotely-sensed estimates of Canadian forest composition to be merged with the greater stand-structural detail obtainable from forest inventory plots. Using species biomass as well as mean annual temperature and total annual precipitation as variables, we performed a nearest neighbour spectral analysis (NNSA) to attribute the FIP showing the smallest Euclidean distance to each 250-m grid cell. Grid cells and FIP were binned into 20-yr age classes; NNSA imputations were then conducted separately for each age class group to ensure that the Euclidean distance between the FIP and the 250-m cell could be attributed more closely to site productivity rather than stand age. Using this technique, we aimed to populate initial LANDIS-II forest landscapes so that they resembled actual forest conditions in the early 2000s. Grid cells with less than 50% forest cover (by area, according to the inventories) were excluded from the simulations. A total of 89 landtypes were created. Further details can be found in Boulanger et al. [19].

#### PICUS

LANDIS-II requires specific, dynamic input parameters to simulate the effect of climate change on forests, which are generally derived from more ecologically detailed, fine-scale forest stand models. Here, we used PICUS version 1.5 [62], which is an individual tree-based, spatially explicit, forest gap model, to develop the dynamic input parameters required to initialize LANDIS-II. PICUS simulates the germination, establishment, growth, and mortality of individual trees in 100-m^2^ gaps or “patches” of forest area. Generally, 100 of these patches are simulated simultaneously as interacting square cells on a contiguous 10 x 10 grid, corresponding to a 1-ha plot sample of forest stand. PICUS runs on annual time steps and accounts for spatially explicit interactions among patches via a 3D canopy light module, and simulates seed dispersal explicitly, as well as the effects of climate and soil properties on tree germination and growth.

To develop the dynamic input parameters for LANDIS-II, we used PICUS to simulate mono-specific stands of each tree species for all landtypes under four different climate forcing scenarios (baseline, RCP 2.6, RCP 4.5, and RCP 8.5) and for specific periods (2000–2010, 2011–2040, 2041–2070, 2071–2100), for a total of 15,130 PICUS simulations. Each PICUS simulation was run at an annual timesteps for 300 yr, assuming a stable climate based on the given forcing scenario and 30-yr period. Specific climate and soil details pertaining to a given LANDIS-II landtype were used as inputs for the PICUS simulations. Tree species in PICUS simulations were parameterized (Table 1) as described in Taylor et al. [63]. A complete description of the calibration and validation procedures for these parameters, along with visualization of the PICUS simulations and resulting LANDIS-II parameters specific to the current study area, can be found in S2 Appendix. More details on the derivation of the dynamic input parameters for LANDIS-II from the PICUS outputs are described below.

**Table 1:** Select input parameters specific to PICUS for species simulated within the study area.

Species	Species code	Soil nitrogen*	Minimum soil pH†	Maximum soil pH†	Minimum GDD (Base temp 5°C) ‡	Maximum GDD (Base temp 5°C) ‡	Maximum SMI§	Optimum SMI§
*Abies balsamea*	ABIE.BAL	2	2	9	150	2723	0.3	0
*Acer rubrum*	ACER.RUB	2	2	9.5	500	6608	0.5	0.05
*Acer saccharum*	ACER.SAH	2	1.7	9.9	450	5093	0.3	0
*Betula alleghaniensis*	BETU.ALL	2	2	10	500	4517	0.5	0.05
*Betula papyrifera*	BETU.PAP	2	2.2	9.4	150	3081	0.5	0.05
*Fagus grandifolia*	FAGU.GRA	2	2.1	9	500	5602	0.7	0.1
*Larix laricina*	LARI.LAR	1	3	9.6	150	2548	0.3	0
*Picea glauca*	PICE.GLA	3	2	10.2	150	2495	0.5	0.05
*Picea mariana*	PICE.MAR	2	2	8.5	150	2495	0.3	0
*Picea rubens*	PICE.RUB	2	2	7.8	450	3239	0.3	0
*Pinus banksiana*	PINU.BAN	1	2.5	10.2	300	3188	0.7	0.1
*Pinus resinosa*	PINU.RES	1	2.5	8	500	3300	0.7	0.1
*Pinus strobus*	PINU.STR	2	2	9.3	500	4261	0.7	0.1
*Populus tremuloides*	POPU.TRE	2	2.3	11	150	3024	0.5	0.05
*Quercus rubra*	QUER.RUB	1	2.3	9.3	500	5171	0.3	0
*Thuja occidentalis*	THUJ.OCC	2	3	10	500	3383	0.7	0.1
*Tsuga canadensis*	TSUG.CAN	2	2.2	9	500	4660	0.5	0.05
Références			**[64–65]**		**[66–67]**		**[68]**	

* Nitrogen response curves: Three classes (1–3), with 1 being very tolerant;

† USDA’s plant database [64] and the Ontario Silvics Manual [65] were used to derive the widest optimum pH range possible;

‡ Growing Degree-Days (GDD). We used McKenney et al.’s [66] growing season model, specifically minimum GDD for the 0°C growing season window with degree-days over 5°C. For the maximum GDD, we used GDD Maximum from McKenney's et al. [67] previous growing season model;

§ Soil Moisture Index (SMI). Determines each species tolerance to drought (see [68], p. 52). HighTolerance (0.1 to 0.7), MedTolerance (0.05 to 0.5), LowTolerance (0 to 0.3).

#### Forest succession and species’ growth potential

Forest succession was simulated using a version of the LANDIS-II Biomass Succession extension v 3.1 [34] modified to account for strict serotiny in jack pine. This extension emulates succession at the stand (grid cell) level by simulating the recruitment and growth of tree cohorts (not individual trees). Multiple cohorts of tree species may establish themselves within each grid cell and interact with each other through resource (i.e., growing space) limitations based on species-specific traits. Succession in each cell is driven by these stand-level interactions, in addition to disturbance history and seed source availability.

Specific parameters that define basic life-history traits were assigned to all species (cf. Table 2 for a full listing). These parameters were homogeneous throughout the entire landscape and kept constant during the simulation. Parameters were collected from various sources (e.g., [69–70]), including expert judgment when empirical information was unavailable. Species- and landtype-specific response parameters for the Biomass Succession extension (namely species establishment probabilities [SEP], maximum annual net primary productivity [maxANPP] and maximum AGB [maxAGB], see below) were simulated for LANDIS-II using PICUS. These three parameters (often referred to as *dynamic inputs* in the LANDIS-II literature) can be updated during a LANDIS-II simulation to account for the effects of climate change.

**Table 2:** LANDIS-II input data for tree species simulated within the study area.

Species code	Longevity	Age at maturity	Shade tolerance†	Effective seed dispersal (m)‡	Maximum seed dispersal (m)	Vegetative regeneration	Post-fire regeneration	Growth curve shape parameter	Mortality curve shape parameter	SEP* (mean ± SD)
ABIE.BAL	150	30	5	25	160	No	None	0	25	0.48±0.05
ACER.RUB	150	10	3	100	200	Yes	Resprout	0	25	0.31±0.21
ACER.SAH	300	40	5	100	200	Yes	Resprout	1	15	0.30±0.14
BETU.ALL	300	40	3	100	400	Yes	Resprout	1	15	0.29±0.19
BETU.PAP	150	20	2	200	5000	Yes	Resprout	0	25	0.55±0.05
FAGU.GRA	250	40	5	30	3000	Yes	None	1	15	0.27±.014
LARI.LAR	150	40	1	50	200	No	None	0	25	0.54±0.06
PICE.GLA	200	30	3	100	303	No	None	1	15	0.43±.041
PICE.MAR	200	30	4	80	200	No	Serotiny	1	15	0.36±0.04
PICE.RUB	300	30	4	100	303	No	None	1	15	0.25±0.11
PINU.BAN	150	20	1	30	100	No	Serotiny	0	25	0.54±0.09
PINU.RES	200	40	2	12	275	No	None	1	15	0.32±0.20
PINU.STR	300	20	3	100	250	No	None	1	15	0.30±0.19
POPU.TRE	150	20	1	1000	5000	Yes	Resprout	0	25	0.59±0.07
QUER.RUB	250	30	3	30	3000	Yes	Resprout	1	15	0.28±0.15
THUJ.OCC	300	30	5	45	60	No	None	1	15	0.26±0.12
TSUG.CAN	300	60	5	30	100	No	None	1	15	0.21±0.11

† Index of the ability of species to establish under varying light levels, where 1 is the least shade tolerant and 5 is the most shade tolerant.

‡ Distance within which 95% of seeds disperse.

*SEP (Species Establishment Probability): Mean and standard deviation values are reported for all landtypes under the baseline climate. More results about SEP and other Biomass Succession dynamic inputs can be found in S2 Appendix.

In the LANDIS-II Biomass Succession extension, the carrying capacity of a given landtype is defined by the maxAGB. This value was set by averaging total AGB of a stand simulated by PICUS after it has reached a stable biomass state, i.e., following the early growth phase of stand development. maxANPP can only be achieved under free growing conditions, i.e. in the total absence of inter-specific competition. To derive maxANPP from the PICUS outputs, we calculated current annual increments (kg·ha^-1^·yr^-1^) over the entire simulation period, which is calculated as total AGB of all trees ≥ 1.3 m tall at the end of each year plus any biomass removed that year by mortality, minus total AGB calculated at the end of the previous year. Maximum values were typically observed during the early stages of stand development (see S2 Appendix). SEP is defined as the probability of a given species’ cohort to successfully establish itself on a given landtype during one time step, under ideal conditions (i.e., when seeds are available and light conditions are adequate), and it can range from 0 to 1. We considered the time necessary for stems >1.3 m tall to accumulate AGB (t) in PICUS as the average result of a random process associated with a constant annual probability of 1/t. We thus simulated the probability of successful establishment of a cohort as a Bernouilli trial conducted every year during a 5-yr time step, i.e., the probability of at least one success in five consecutive trials based on binomial distribution.

#### Bias correction of PICUS outputs and verification of emerging LANDIS-II successional patterns

Succession patterns under the baseline climate scenario were visualized and qualitatively verified (see S3 Appendix) against those reported in the literature (e.g., [71, 72]). Accordingly, some adjustments were made to the species-specific static growth and mortality curve shape parameters (Table 2), which determine the acceleration of each species’ growth rate and how soon mortality begins as each cohort of species reaches its maximum longevity. Additional details about this procedure can be found in S3 Appendix.

Finally, we validated the dynamic inputs obtained from PICUS by comparing the initial AGB of each species produced during the spin-up phase of LANDIS-II under the baseline climate scenario against the biomass reported in the NFI forest cover maps [50]. These biomass values sometimes showed substantial bias that was minimized by a two-stage correction procedure. First, maxAGB and maxANNP parameters for all species other than balsam fir were multiplied by a single scalar to adjust average initial total biomass within the simulated landscape to agree with landscape-scale biomass densities reported in the NFI cover maps. Second, simulations for balsam fir required special treatment because of a previously documented over-sensitivity to limited water availability [63], which caused balsam fir biomass to be systematically underestimated. This was adjusted by multiplying maxAGB and maxANNP for balsam fir by a larger scalar. Additional details and illustrations can be found in S3 Appendix.

#### Natural disturbances

Fire and spruce budworm (SBW, *Choristoneura fumiferana* [Clem.]) outbreaks are the two disturbance agents responsible for most of the natural disturbances in the study area [56]; both are widely recognized to have major impacts on Canada’s forest landscapes [73].

All disturbance regimes, historical (baseline) and projected, have been quantified in previous published studies [56, 74]. In our LANDIS-II simulations, fire effects were captured using the LANDIS-II Base Fire extension [75], which simulates stochastic fire events dependent upon fire ignition, initiation and spread. Fire regime data (annual area burned, fire occurrence, and mean fire size) were first summarized into ‘‘fire regions” corresponding to the intersection of the study region with the Canadian Homogeneous Fire Regime (HFR) zones of Boulanger et al. [74]. Baseline and future fire regime parameters within each fire region were calibrated according to models developed by Boulanger et al. [74] and further updated for different RCP scenarios [18].

Outbreaks of SBW were simulated using the LANDIS-II Biological Disturbance Agent (BDA) extension v3.0 [76–77], which is specifically designed to simulate host tree mortality following insect outbreaks. Host tree species for SBW included, from the most to the least vulnerable: balsam fir, white spruce, red spruce and black spruce. Outbreaks are simulated as probabilistic events at the cell level with probabilities being a function of site and neighbourhood resource dominance (e.g., host abundance within a 1-km radius of the cell) as well as regional outbreak status. Outbreak impacts (tree mortality) are contingent on these probabilities as well as on host species’ and age-specific susceptibility. Parameters used in this study were obtained from various sources for the mixed boreal forest [78, 79]. Regional outbreaks were calibrated at the highest severity level possible using this extension and were set to last 10 yr at most and to occur every 35 yr, in accordance with observed typical regional recurrence cycles [80]. Simulation parameters for both natural disturbances can be found in S4 Appendix.

#### Forest harvesting

Forest harvesting was simulated using the Biomass Harvest extension (v3.0; [81]). Historical harvest data (harvested AGB) were retrieved from Québec’s provincial records for the 1980–2000 period (Gouvernment du Québec, unpublished data). Mean harvested patch size and total harvested area were summarized by “management areas”, i.e., either by forest management units for public lands (87.3% of the study area) or by ecodistricts [82] for private lands and for lands located north of the northern limits of merchantable harvesting (12.7% of the study area). Only clearcuts were simulated on public lands as this logging strategy is the most frequently used in the study area [55]. On private lands, we used a national map of forest disturbances derived from MODIS imagery [56] to simulate biomass removed by harvesting during 2002–2011. Prescriptions on private lands included low-level partial harvesting (1–40% of biomass removed), high-level partial harvesting (41–80% biomass removed) and clearcutting. In the simulations, clearcutting was represented by the removal of all age cohorts, except for the 0–5 yr cohort. Only stands that contained tree cohorts older than 60 yr were allowed to be harvested. Harvesting parameters were set so that a constant area was harvested throughout the simulations, unless there was not enough stands older than 60 yr. Additional details about projections of disturbance regime can be found in S4 Appendix.

### Simulation design

#### Cumulative impacts

To estimate the cumulative impacts of harvesting and climate change on BBWO productivity, we ran simulations in which successional dynamics as well as all disturbances (wildfire, spruce budworm and forest harvesting) were included. Five replicates were run for 100 yr, starting in the year 2000, under each climate forcing scenario with 5-yr time steps. Except for scenarios involving the baseline climate, climate-sensitive parameters (fire regime, maxANPP, maxAGB and SEP) were allowed to change in 2010, 2040 and 2070, according to the average climate corresponding to each forcing scenario.

#### Relative importance of stand-scale processes, fire regime and harvesting

We ran additional simulations according to a full, three-way factorial design to assess the specific importance of harvesting as well as climate-induced changes in stand-scale processes and fire on BBWO productivity. The effects of stand-level drivers (i.e., climate effects on SEP, maxANPP and maxAGB) were tested either by keeping these parameters constant, according to baseline calibration conditions, or by updating them according to the respective RCP scenarios. Similarly, sensitivity to fire effects was tested by including or omitting the impacts of climate change on the fire regime, where fire parameters calibrated for the baseline period were either kept constant throughout the simulation period or were updated according to the projections of Boulanger et al. [74]. The effect of harvesting was tested by including or omitting this disturbance in the simulations. This full three-way factorial design was repeated for each of the three RCP scenarios. Five replicates were run for each set of simulations under each forcing scenario for a total of 120 simulations (5 replicates * 2 harvesting levels [no harvesting, full harvesting] * 2 fire levels [baseline fire level, projected fire level] * 2 stand-scale levels [baseline and projected dynamic Biomass Succession parameters] * 3 RCP scenarios [RCP 2.6, RCP 4.5, RCP 8.5]). The impacts of SBW outbreaks were included as a “background” disturbance in all simulations.

#### BBWO habitat classification and potential productivity

Based on recent studies of habitat selection and potential productivity of BBWO in central Québec [38, 39, 42], the LANDIS-II grid cell outputs were reclassified into six habitat types (Table 3). To ensure that BBWO potential productivity was estimated in a spatially-explicit manner, we used the *AREA* function in FRAGSTATS (v 4.2.1.603; [83]) to aggregate spatially contiguous pixels of the same habitat into patches that were equal to, or larger than, the mean BBWO home range size for a given habitat type as assessed from the literature (Table 3).

**Table 3:** Forest stand characteristics of BBWO habitat type, mean home range size, and mean productivity per home range in each habitat type.

Habitat type	Forest stand characteristics		Mean homerange size (ha)^a^	Mean productivity (no. of fledglings per year)^b^
	Age	Year post-fire		
Old coniferous unburned forest	≥80 yr	-	150	1.5
Old mixed unburned forest	≥80 yr	-	300	1.0
Recently burned old coniferous forest	≥80 yr	1–5	40	1.4
Recently burned young coniferous forest	<80 yr	1–5	100	0.25
Older burned coniferous forest	≥80 yr	6–10	200	0.4

^a^ Based on [38, 42, 43].

^b^ Based on [39, 42, 43].

For all aggregated patches of each habitat type, BBWO potential productivity was estimated as the theoretical number of young produced, based on mean productivity for each habitat type (Table 3) multiplied by the number of patches. We defined total BBWO productivity as all fledglings that could be produced in the study area, for all habitat types combined. The relative percentage contribution of each habitat type was then estimated by dividing its potential productivity by the total potential productivity over a given period (relative contribution of a habitat type = potential productivity of this habitat type/total productivity).

### Analyses

#### Cumulative impacts

The cumulative impacts of harvesting and climate change were assessed by comparing temporal trends in the following simulated variables: 1) specific tree AGB; 2) total BBWO potential productivity under each climate scenario; 3) BBWO potential productivity in each habitat type; and 4) relative contributions of the three most important habitat types estimated for each climate scenario. For each climate scenario, results for these variables were obtained by averaging outputs [either tree AGB or BBWO habitat-related variables] from the five replicates of the simulations used to assess the cumulative impacts for each climate scenario.

#### Importance of stand-scale processes, fire and harvesting

The relative importance of each driver of change on BBWO habitat was assessed using omega-squared values (ω^2^), which is an estimate of the variance of the dependent variable that can be explained by the driver of change. In this case, the dependent variable was total BBWO potential productivity for the entire study area. Following a three-way factorial ANOVA, where each driver of change (stand-scale, fire regime, harvesting) was considered as a factor, we calculated ω^2^ for each driver of change, at each time step, as:

$$\omega^{2}=\left[SS_{effect}-\left(df_{effect}\right)*\left(MS_{error}\right)\right]/\left[MS_{error}+SS_{tot}\right]$$

where SS_effect_ is the sum of squares related to the driver of change (the effect), df_effect_ is the degree of freedom of the effect, MS_error_ is the mean square of the error, and SS_tot_ is the total sum of squares. ANOVA and ω^2^ calculations were performed separately for each RCP scenario.

In addition to the proportion of variance explained, we also sought to estimate the quantitative impact of each driver of change on BBWO potential productivity, while controlling for all other factors [30]. To do so, impacts were assessed through sensitivity analyses by calculating ΔProd, which is the percentage difference in simulated BBWO total potential productivity at the regional level (i.e., study area) between the simulations considering all cumulative impacts (i.e., the “full” model) and corresponding simulations omitting the driver of interest (i.e., the “reduced” model) (see below), for a given time step (*t*) and forcing scenario. Reduced model testing for impacts of changes in fire and in stand-scale drivers used simulations where fire regime and SEP / maxANPP / maxAGB parameters, respectively, were calibrated for the baseline climate. To assess the quantitative impacts of harvesting, the reduced model used simulations that simply omitted harvesting while controlling for all other factors.

We also tested the impacts of each driver of change on each tree species. Percentage differences in simulated AGB (ΔB) between simulations run under the full model and those run under each reduced model were calculated at each time step for each species. These analyses are similar to those conducted by Boulanger et al. [30]. All pre- and post-model data processing and analyses were conducted using R version 3.3.0 [84].

## Results

### Cumulative impacts of climate change and harvesting on forest conditions

**Fig 2:**
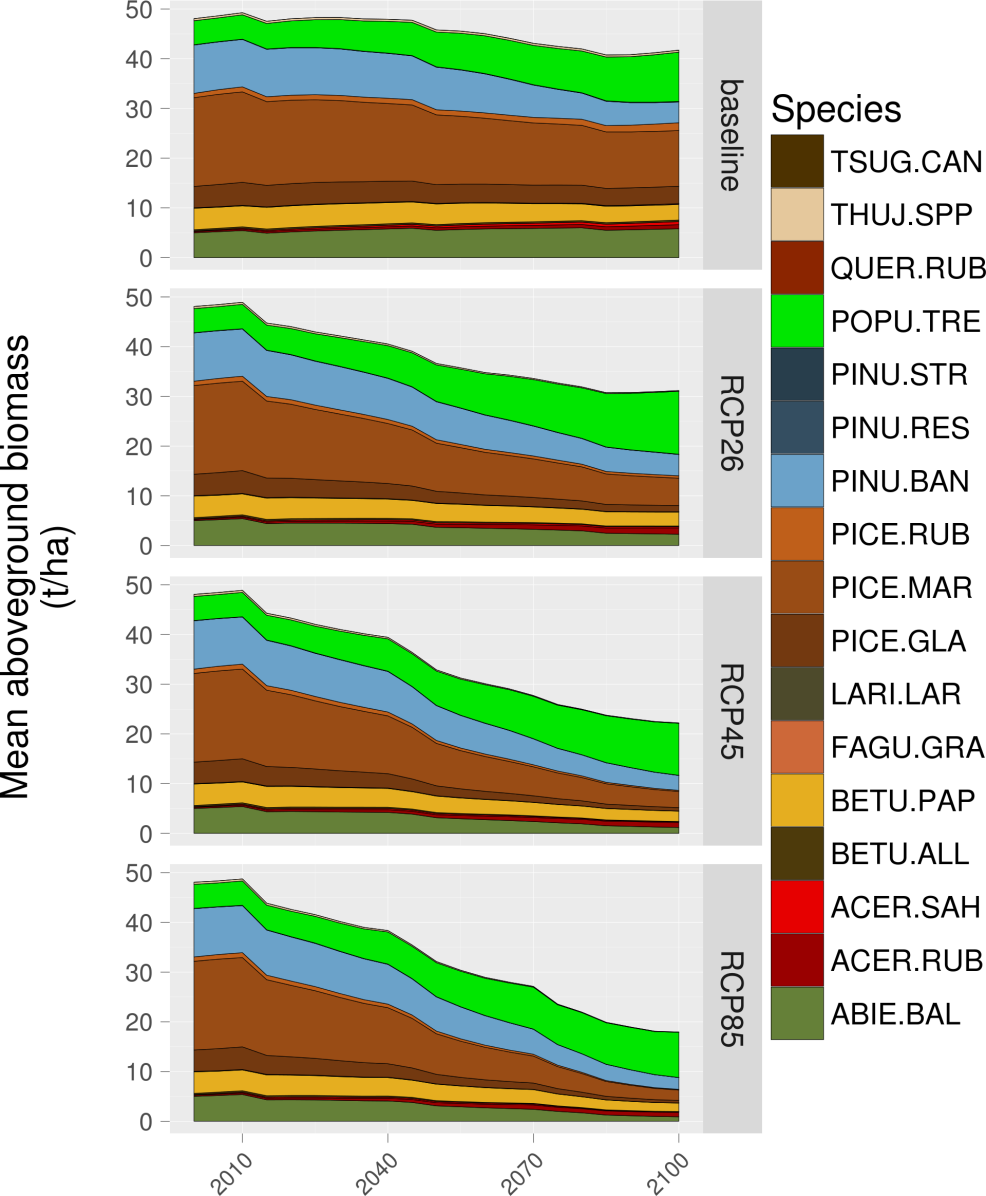
Projected cumulated changes in tree species AGB under baseline, RCP 2.6, RCP 4.5 and RCP 8.5 climate scenarios.

Total AGB was projected to decrease slightly over time in the study area under the baseline climate scenario (from 48.1 t/ha in 2000 to 42.1 t/ha in 2100; Fig 2). Under the baseline scenario, trembling aspen showed a large increase in biomass (from 4.8 t/ha in 2000 to 13.1 t/ha in 2100) whereas black spruce showed a large decrease (from 24.2 t/ha in 2000 to 7.5 t/ha in 2100). Total AGB continually decreased as projected warming proceeded, with RCP 8.5 driving the largest (most rapid) reductions (Fig 2). Overall, compared to initial conditions, total AGB decreased by 32.8%, 52.4% and 65.0% over the entire simulation period under the RCP 2.6, 4.5 and 8.5 scenarios, respectively. Coniferous tree species, notably balsam fir and black spruce, were most negatively impacted by climate forcing with reductions in biomass of 82.9% and 92.6%, respectively, while trembling aspen and red maple projections showed increases of 84% and 455%, respectively, under RCP 8.5. Absolute changes in biomass of other species were minor, regardless of the forcing scenario, largely because their simulated abundance was low throughout all simulations.

Most of the changes in tree species biomass under stronger anthropogenic climate forcing would result from changes in the fire regime (see S5 Appendix). Increasing fire activity would be detrimental for the AGB of virtually all tree species, with changes in AGB (ΔB) exceeding -50% for the majority of species by 2100 under RCP 8.5. Trembling aspen appears to be the only species for which large increases in fire activity would not noticeably affect AGB (S5 Appendix). The detrimental impacts of harvesting would also be rather important for many species except trembling aspen, balsam fir, white birch and red maple, regardless of climate forcing. Aside from these four species, harvesting would reduce AGB by ca. 25–50% by 2100. Climate-induced changes in stand-level processes would be generally negligible or positive for most boreal species in the study area, except for black spruce, for which an increasing warming would decrease ΔB by >30% by 2100 under RCP 8.5. Except for balsam fir, black spruce, jack pine and trembling aspen, moderate (RCP 2.6, RCP 4.5) anthropogenic climate forcing was projected to actually increase ΔB. Stronger positive impacts of climate-induced changes in stand-level processes on ΔB would occur under RCP 8.5 for temperate deciduous species, including red and sugar maples, beech and red oak (S5 Appendix).

### Cumulative impacts of climate change and harvesting on BBWO habitats and potential productivity

**Fig 3:**
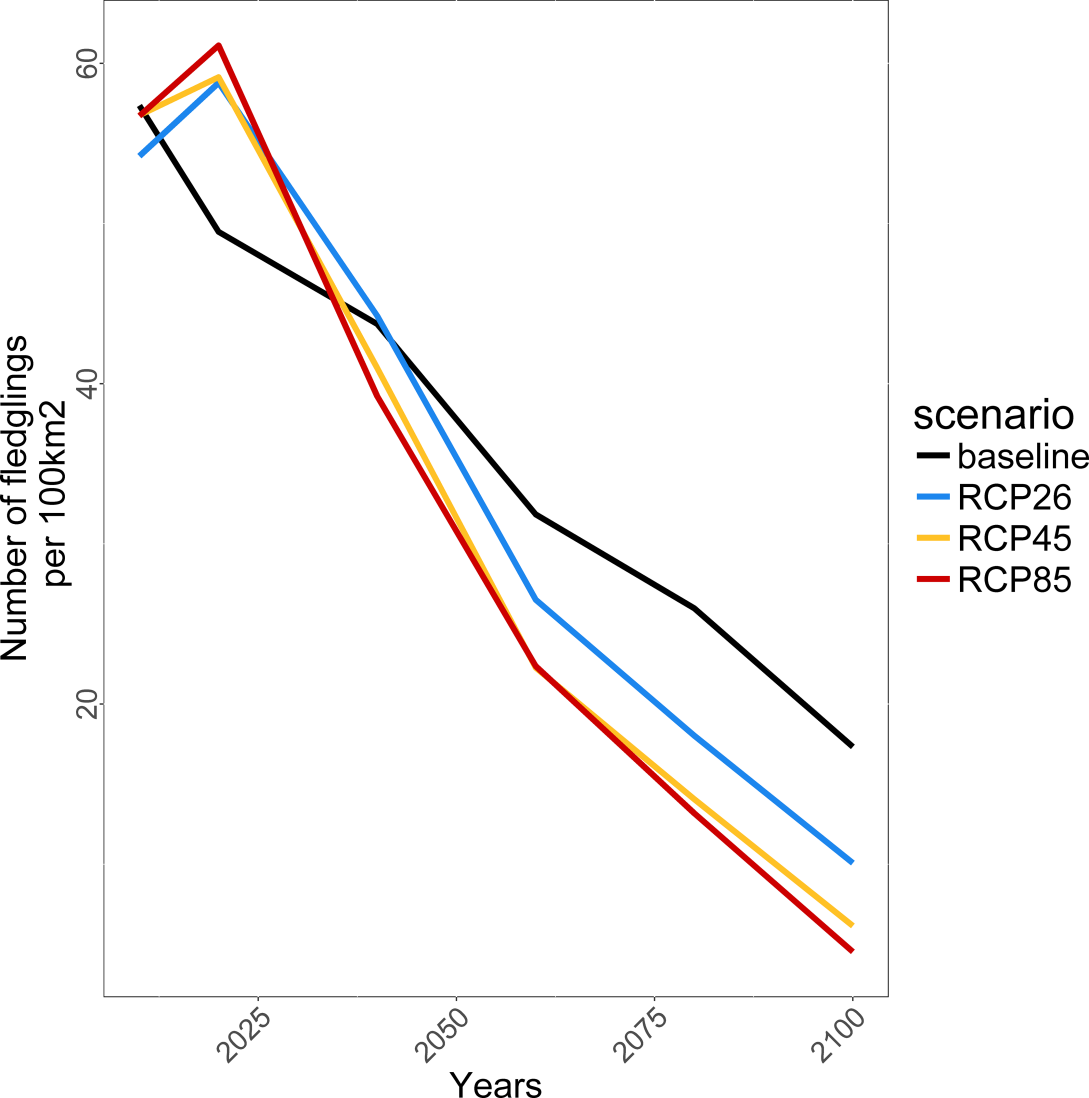
Total Black-backed Woodpecker productivity in the study area as projected for the baseline, RCP 2.6, RCP 4.5 and RCP 8.5 climate scenarios.

Under initial conditions (year 2000), the total potential productivity of BBWO was estimated to be ca. 54–58 fledglings/100 km^2^/yr in the study area. BBWO potential productivity was projected to decrease under all climate scenarios, including the baseline scenario (Fig 3), although differences among RCP scenarios were relatively small in 2100 (<5 fledglings/100 km^2^). Higher BBWO productivity was projected in the early part of the 21st century with projected climate warming (compared to the baseline scenario) until ca. 2020, becoming lower thereafter (Fig 3). By 2100, however, total potential productivity relative to initial conditions was projected to decrease by 69%, 81%, 89%, and 92% under the baseline, RCP 2.6, RCP 4.5 and RCP 8.5 scenarios, respectively.

**Fig 4:**
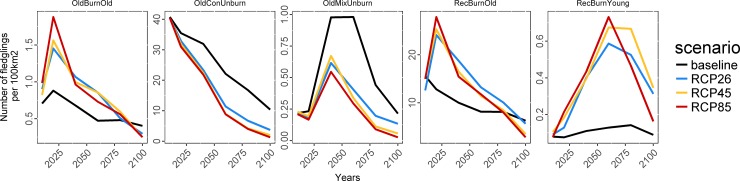
Comparison of projected Black-backed Woodpecker productivity (number of fledglings/100 km^2^) for each habitat type under the baseline, RCP 2.6, RCP 4.5, and RCP 8.5 climate scenarios.

**Fig 5:**
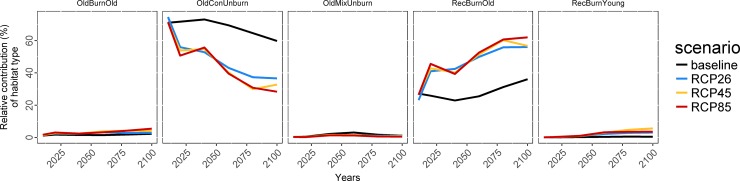
Relative contribution of habitat types to total Black-backed Woodpecker productivity as projected for the baseline, RCP 2.6, RCP 4.5 and RCP 8.5 climate scenarios.

The projected number of fledglings produced in each habitat type varied throughout the simulation period. Recently burned old coniferous stands, old coniferous unburned stands, and older burned coniferous stands produced the most BBWO fledglings, regardless of the climate scenario, while production of fledglings in old mixed unburned coniferous stands and recently burned young stands was very low (Fig 4). Under the RCP climate forcing scenarios, BBWO potential productivity in recently burned old coniferous stands increased rapidly, from ca. 14 fledglings/100 km^2^/yr in 2010 to ca. 25–30 by 2020, before declining drastically to ≤6 fledglings/100 km^2^/yr in 2100 (RCP 4.5 and RCP 8.5; Fig 4). Despite these variations, the relative contribution of recently burned old coniferous stands increased throughout the simulation period under each climatic scenario, and reached approximately 56% (RCP 4.5) to 62% (RCP 8.5) of the total production of BBWO fledglings in 2100 (Fig 5). Old coniferous unburned stands, the most productive habitat type for BBWO at the beginning of the simulation period, showed striking declines under each forcing scenario from ca. 40 fledglings/100 km^2^/yr down to <4 fledglings/100 km^2^/yr in 2100 for all forcing scenarios (Fig 4). At the beginning of the simulated period, old coniferous unburned stands represented ca. 70–75% of the total production of BBWO fledglings, but this was projected to decrease to only 28 and 33% for RCP 8.5 and RCP 4.5, respectively (Fig 5). The total production in old coniferous unburned stands would remain approximately twice as high under the baseline scenario than under increasing anthropogenic climate forcing. The relative contribution of older burned coniferous stands and old mixed unburned coniferous stands to total BBWO production was relatively small during the entire simulation period, with a maximum of ca. 5% and ca. 0.5% in 2100, respectively (Fig 5).

Under the baseline climate scenario, old coniferous unburned stands were projected to remain the most regionally productive habitat type for BBWO (between ca. 60–71% of total fledglings produced). However, shifts were projected by ca. 2050 under increasing anthropogenic climate forcing, where recently burned old coniferous stands were projected to become more productive than old coniferous unburned stands, with the differences being amplified under the more severe climate forcing scenarios (Fig 5).

### Relative importance of climate-induced changes in stand- and landscape-scale drivers for BBWO productivity

**Fig 6:**
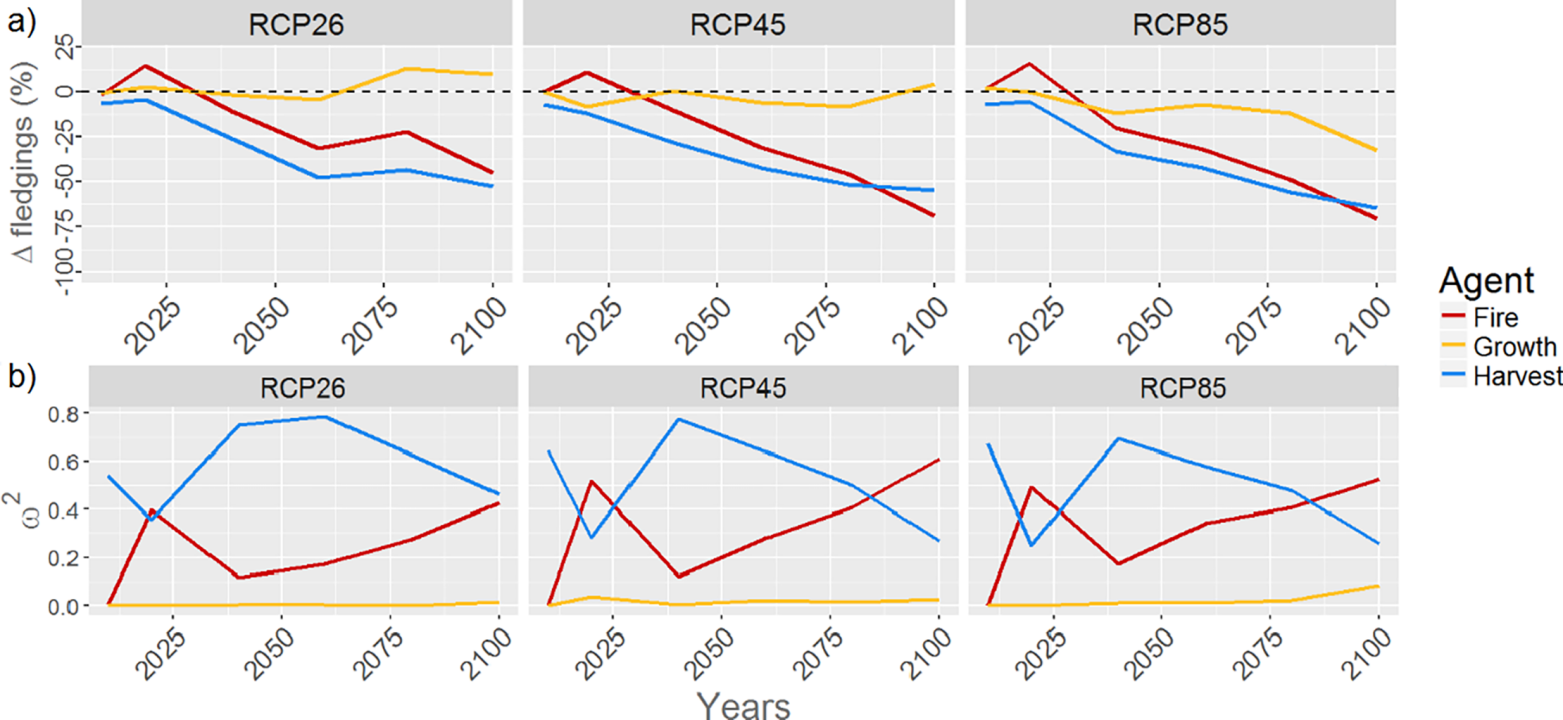
Trends in a) the relative difference of potential productivity (ΔProd) between the reduced and the full model for each driver of change and in b) ω^2^ for each driver of change in Black-backed Woodpecker potential productivity under the RCP 2.6, RCP 4.5 and RCP 8.5 forcing scenarios. In b), ω^2^ values were obtained through three-way factorial ANOVA performed at each time step.

Harvesting was projected to reduce BBWO potential productivity throughout the simulation period (Fig 6A), and it was generally was the most important single driver of change under all RCP scenarios (Fig 6B). Indeed, harvesting ranked as the second most important driver of change only in 2020 and after 2080 under RCP 4.5 and RCP 8.5 while being most important under all other simulated conditions. Regardless of climate forcing, changes in fire regime were initially projected to increase BBWO potential productivity by 20–25% in 2020, but thereafter, further changes in the fire regime had negative impacts, reaching rather similar values to those reported for harvesting by 2100 (Fig 6A). Climate-induced changes in stand-scale drivers had minimal impacts on BBWO potential productivity throughout the simulation period compared with harvesting or climate-induced changes in fire disturbance (Fig 6).

## Discussion

Few studies have projected the cumulative impacts of climate change and harvesting at both the stand and landscape levels on bird habitats using a spatially explicit forest landscape models (but see [85, 86]). Our study attempted to provide a more realistic estimation of the cumulative impacts of climate change and harvesting on the habitat and productivity of a focal boreal bird species. Our results demonstrate these impacts are likely to be detrimental for the BBWO with a projected decline of 81 to 92% in potential productivity by 2100.

Our simulations suggest that the cumulative impacts of forest harvesting and climate change on the forest ecosystem will cause major reductions in the availability of future BBWO habitat, with the potential productivity of this focal species being more severely reduced under stronger anthropogenic climate forcing. Among other impacts, climate change would impose changes in the relative importance of available habitat types for BBWO potential productivity. Under the baseline climate scenario, populations were largely maintained by the productivity of old coniferous stands. With climate warming, however, recently burned old coniferous forests would likely become more important for supporting BBWO. Conversely, increased reliance on burned forests under a warmer climate would not completely compensate for decreases in productivity due to the loss of old-growth coniferous forest, notably to harvesting. As a consequence, the projected potential productivity of BBWO by 2100 (reduced to below <10 fledglings/100 km^2^/yr) may not be high enough to support sustainable populations under any realistic anthropogenic forcing scenario. Based on historical forest age-structure, BBWO productivity was estimated to be ca. 75 fledglings/100 km^2^/yr in a 2,500-km^2^ boreal landscape in central Québec [39]. Although we cannot estimate the carrying capacity or population growth index from our simulations (see section below), the major declines in the potential number of fledglings projected for all climate warming scenarios (85–93% decrease) highlight a major threat to the long-term sustainability of the population (*sensu* [87]).

Nevertheless, our results show that important declines in BBWO potential productivity would likely occur in this area even under present-day climate, underlining the importance of non climatically-driven changes. Harvesting was found to be the most important driver of changes in BBWO potential productivity, regardless of the anthropogenic forcing scenario. In this context, harvesting would interact synergistically with climate-induced changes in forest landscapes to further shrink high-quality BBWO habitats over the long term. This striking result was contrary to our expectations as recent analyses [30] have indicated that harvesting should have relatively little impact, compared to changes in the fire regime and in stand-scale processes, in projected future southern boreal forest landscapes in eastern Canada. These unexpected results might be explained by the fact that harvesting rates in the study area are rather high (between 0.4–0.8%.yr^-1^ for most forest management units) when compared with those reported by Boulanger et al. [19] when they simulated a southern part of the eastern boreal forest in which harvesting had been less active compared with the northern region over the last decades [55]. Moreover, the estimation of BBWO potential productivity relied significantly on specific age-defined stand types (with the most productive stand types being associated with old-growth forests, either burned or not), a feature that is not directly considered when assessing the relative impacts of harvesting and climate change on tree species biomass *per se*. By targeting old-growth stands, high business-as-usual logging rates would likely contribute to the decline of suitable late-successional coniferous stands, while maintaining some important deciduous species, including trembling aspen, white birch and red maple, regardless of the climate scenarios. Boucher et al. [55] have shown that high harvesting pressure has historically contributed to marked decreases in the abundance of old-growth stands and increases in pioneer, deciduous stands in Québec’s boreal forest. General decreases in old-growth stands due to high harvesting pressure might explain, to a large extent, the decrease in potential number of fledglings, even under baseline climate conditions. Silvicultural practices, as well as natural disturbances, have been found to play an important role in defining how landscapes will respond to climate change (e.g., [88–89]), notably by providing new opportunities for climate-adapted species to invade a site [90]. Our 100-yr simulations did not reveal increasing impacts of harvesting along with increasing climate forcing. However, we can expect that the interacting impacts of harvesting and climate change might worsen beyond 2100, as harvesting would increasingly favour the recruitment of warmer-adapted deciduous species under increased climate forcing, and reduce the supply of suitable habitats dominated by boreal conifers. Longer simulations would be needed to explore these interactions on future boreal bird habitats.

Despite the major impacts of harvesting, climate-driven changes in BBWO productivity will be very significant and mostly linked to changes in the fire regime. Fire seasons in the study area have been projected to lengthen, leading to more frequent and severe fire-conducive weather [74]. BBWO is a disturbance-adapted species [39, 42, 43], and it is known to respond positively to natural disturbances in boreal forests, especially wildfires [43]. However, we found that large increases in area burned, especially under “worst-case” climate forcing (i.e., RCP 8.5), would be detrimental to BBWO productivity. As such, the negative impacts of a climate-induced increase in fire activity might look contradictory. Negative impacts would be the result of a very sharp long-term increase in fire activity, cancelling the short-term positive impacts of increased area burned. Impacts of long-term increases in fire activity on BBWO habitats would likely be two-fold. First, increased area burned would lower the mean forest stand age, thus reducing the availability of large patches of highly suitable old forest stands. Sharp increases in fire activity were found to be detrimental by old-growth bird specialists in Alberta [85]. Second, increased burning rates would promote the regeneration/re-sprouting of pioneer deciduous species (i.e., white birch, trembling aspen and red maple) at the expense of late-successional conifers (i.e., balsam fir, black spruce), further decreasing the availability of highly suitable old coniferous stands and those subsequently available for burning. Previous simulations performed on southern boreal landscapes [19] also showed that very short fire return intervals (< 50 yr), as projected for the RCP 8.5 radiative forcing by 2070 in the study area [19], would almost certainly cause the widespread decline of boreal late-successional fire-avoiders such as white spruce and balsam fir [91]. Black spruce forests may also decline rapidly if the fire return interval were to shorten to values close to that of typical seed-bearing age (ca. 30 yr) [92]. Very short fire return intervals, comparable to those projected here (ca. 30 yr under RCP 8.5, see S2), would likely effectively prevent the gradual age-related conversion of pioneer deciduous stands into late-successional coniferous stands, reinforcing long-term decreases in potential fledgling productivity.

Our simulations showed that climate-induced changes in stand-scale processes (e.g., regeneration, growth of trees) are likely to have little impact on future BBWO potential productivity. Previous simulations performed on southern Canadian boreal forests [19, 63] have shown that significant warming (similar to that resulting from the RCP 8.5 forcing scenario) will impose strong constraints on the growth of several conifer species (notably balsam fir, white and black spruces, and larch) while favouring increased productivity of warm-adapted deciduous species (notably red maple) in most of the southern boreal forest. We projected generally smaller impacts of stand-level factors on forest landscapes, and hence on BBWO habitats, than those reported by Boulanger et al. [30], which we attribute to our study area lying further north. Moreover, unlike changes resulting from fire or harvesting [90], climate-induced changes in growth in forest landscapes generally manifest themselves on a longer-term perspective. Such system “inertias” might explain why stand-level drivers had little relative impact on BBWO potential productivity over the relatively short simulated 100-yr period. Our analyses did show that under RCP 8.5, black spruce biomass would strongly decline (>25%), but mostly after 2070. In this context, climate-induced impacts on stand-scale processes might impose increasing constraints on BBWO habitats and, consequently, on BBWO productivity, but over a much longer-term (>100 yr).

### Conservation perspectives

Of greatest importance, our results suggest that maintaining current harvesting strategies could exacerbate the negative impacts of climate change on BBWO habitats and potential productivity. Even without climate forcing, however, business-as-usual harvesting strategies could lead to marked decreases in BBWO productivity. Similar results were also found by Mahon et al. [85] for northeastern Alberta. Woodpecker occurrence has been related to forest bird richness [47, 48], and in using BBWO as an indicator for deadwood and old-growth boreal forests [38, 45, 46], our results suggest that many boreal bird species may be subject to dramatic population decrease due to climate change, and to anthropogenically-induced reductions in mature coniferous forest cover. Based on species distribution models, Stralberg et al. [93] highlight the importance of forest growth limitation and succession on time-lags in bird population responses to climate change under which boreal species associated with mature forests may suffer dramatic reductions in suitable habitat over the next century. Reducing harvesting levels in this area might mitigate the impact of climate-induced decrease in old-growth coniferous stands. Partial-cutting strategies that promote the retention of >50% of the stand cover and old stems, or hindering deciduous regeneration, might also be favoured in this context. Retention of a minimum old-growth forest content upfront during harvest planning might also reduce yearly variations in annual allowable cut due to fire losses [94] and reduce unexpected timber shortfalls under a warming climate [30, 95].

### Limitations of the simulations

Our simulations provide insights into how climate change and forest management practices may affect the future of BBWO in Canada’s eastern boreal forest, but there are some important caveats. First, fire effects on forest landscapes probably were overestimated because vegetation feedbacks were not taken into account. Such feedbacks are likely to be negative [96], with recurrent fires both reducing fuel loads and stimulating the production of less flammable fuel, i.e., young, predominantly deciduous, stands. Second, the impacts of climate change on spruce budworm population dynamics and hence on the vulnerability of spruce budworm host species were not included. Third, the PICUS simulations did not consider the possible effects of CO_2_ fertilization on tree growth and stand succession. The potential interaction of higher CO_2_ with significantly longer growing seasons adds uncertainty to the predictions of reduced forest growth, particularly in regions where soil moisture and nutrients are non-limiting (e.g., [97–98]). Also, given that productivity patterns were calibrated for 30-yr averages of a future climate, our results likely overestimate survival and forest productivity because potential widespread “pulses” of drought-induced dieback (e.g., [12]) were not considered. Moreover, variations in total BBWO potential productivity are not solely dependent upon the processes analyzed in this study. Temporal and spatial variations might also arise from ongoing stand development dictated by initial stand conditions or result from recent fire activity, for instance.

Although our simulations attempt to integrate habitat and demographic information, we did not present landscape-based population viability models (*sensu* [10]), mostly because the detailed and critical demographic information needed in such models (i.e., survival rates and dispersion/colonization rates per habitat types) are not available for BBWO–a problem typical for most boreal bird species. For instance, we did not take into account climatically-driven physiological, behavioural or phenological processes that may modify species’ life history traits and vital rates [10, 99]. In this context, changes in habitat could only provide, as a proxy, a partial assessment of the potential changes in BBWO vital rates through changes in specific resources related to these habitats [10]. Likewise, we did not take into account demographic processes such as density dependence, local abundance, productivity, and dispersal, or complex interactions among birds and their prey, that could further help determine the future abundance of BBWO in conjunction with habitat projections. It is currently unknown how the insectivorous BBWO will be affected by potential climate-induced disruptions to the distribution of its prey species. As ectotherms, it is considered that insects will respond rapidly to shifts in climate conditions (e.g., [20, 100]), although some species may be subject to resource/habitat constraints (e.g., [101]). Furthermore, bird species may exhibit adaptation to climate change [102, 103], although species’ plasticity has been little studied in birds [103, 104].

## Conclusion

Our study provides landscape-scale analyses of climate change on the habitat of a focal bird species in Canada’s eastern boreal forest. Since BBWO is considered an indicator species for recent deadwood and old-growth biodiversity in the boreal forest [38, 45, 46], our results suggest that a large number of species may be greatly altered by the cumulative impacts of forest disturbances (natural and anthropogenic) and climate change in this region. Forest harvesting appeared to be the single most important driver of change, because it reduces the area of old-growth coniferous stands, regardless of the climate scenario. Thus, the results of our study suggest that the conservation of these components of biodiversity may benefit from reduced harvesting levels and management strategies that promote coniferous species.

The challenge we are confronting is how to balance the management of multiple forest values and ecosystem services while reducing uncertainty about the future conditions within which the forest is going to evolve, and projecting future habitats for species like the BBWO is part of that process. Finding convergence and implementing it in strategic forest planning is key to achieving sustainability. In that context, the generalist quality of a forest simulator such as LANDIS-II makes it a useful tool for integrating ecological processes and testing management strategies with multiple simultaneous objectives.

## Supporting information

S1 Appendix Climate projections. (PDF)

S2 Appendix Parameterization of LANDIS-II Biomass Succession dynamic inputs and verification of LANDIS-II Biomass Succession emerging successional pathways. (PDF)

S3 Appendix Bias correction of LANDIS-II Biomass Succession dynamic inputs. (PDF)

S4 Appendix Natural and anthropogenic disturbances as simulated in LANDIS-II. (PDF)

S5 Appendix Trends in species-specific mean biomass differences (ΔB). (PDF)
