# Supplementary material for: Harvesting interacts with climate change to affect future habitat quality of a focal species in eastern Canada’s boreal forest
Source: PLoS One. 2018 Feb 7;13(2):e0191645. doi: 10.1371/journal.pone.0191645 (PMC5802891; doi:10.1371/journal.pone.0191645)
Supplement: S1 Appendix — (PDF) [file pone.0191645.s001.pdf]

S1 Appendix

Climate projections

a) Mean annual temperature

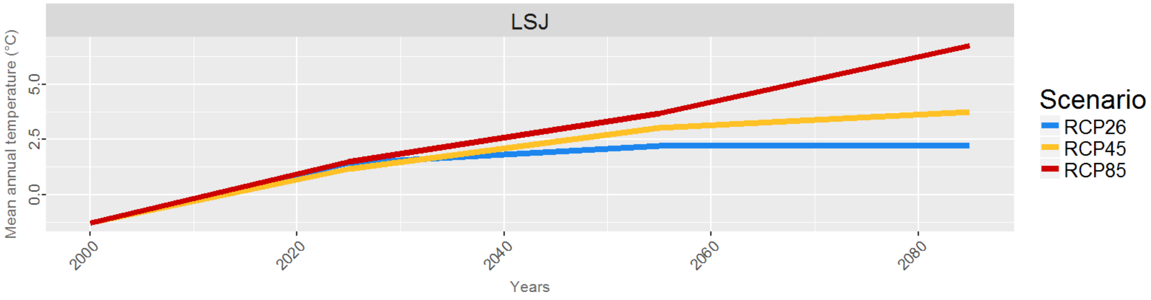

b) Total annual precipitation

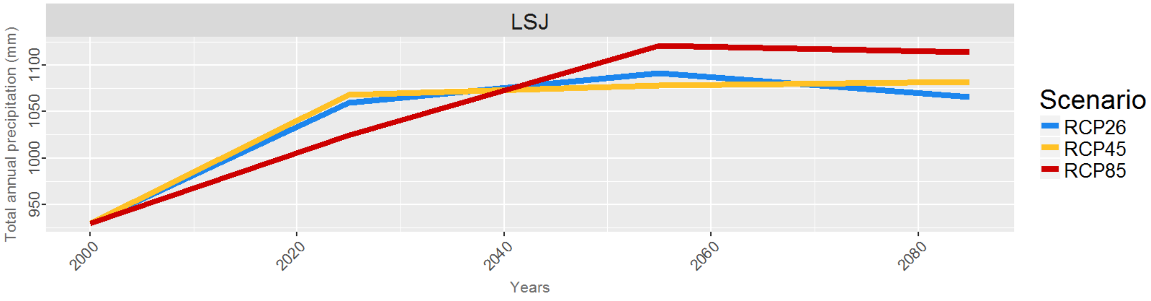

S1 Fig. Current and future projections for a) mean annual temperature (MAT) and b) total annual precipitation from the Canadian Coupled Global Climate Model CanESM2 for each of the three different forcing scenarios i.e. RCP 2.6, RCP 4.5 and RCP 8.5 used in our simulations. Baseline (1981-2010) and future 30-year values were averaged to better reflect the climate data that were used in LANDIS-II to parameterize tree growth.
