# Supplementary material for: Harvesting interacts with climate change to affect future habitat quality of a focal species in eastern Canada’s boreal forest
Source: PLoS One. 2018 Feb 7;13(2):e0191645. doi: 10.1371/journal.pone.0191645 (PMC5802891; doi:10.1371/journal.pone.0191645)
Supplement: S2 Appendix — (PDF) [file pone.0191645.s002.pdf]

S2 Appendix

Parameterization of LANDIS-II Biomass Succession dynamic inputs and verification of LANDIS-II Biomass Succession emerging successional pathways

Parameterization of LANDIS-II Biomass Succession dynamic inputs

Three sets of parameters of the Biomass Succession extension, often referred to as *dynamic inputs* in the LANDIS-II literature, implicitly integrate the physiological response of individual species as a result of environmental conditions. Not only do they usually differ among species and landtypes, but they can also be updated at any time step throughout the course of a simulation to account for the effects of climate change, for instance. They are 1) maximum biomass (maxAGB), 2) maximum aboveground net primary productivity (maxANPP), and 3) species establishment probability (SEP).

These dynamic inputs were derived from PICUS outputs. We used PICUS to simulate ideal conditions where a single cohort of a given species is initiated on bare ground, leading to the development of a monospecific stand. PICUS simulations were conducted at a yearly timestep and run for 300 years. That process was repeated for every combination of tree species, landtypes, climate scenarios (baseline, RCP 2.6, RCP 4.5 and RCP 8.5) and periods (2011-2040, 2041-2070, 2071-2100), for a total of 15,130 PICUS simulations (S2.1 Fig). Specific climate and soil information pertaining to a given landtype were used as inputs for PICUS simulations. Tree species in PICUS simulations were parameterized as described in Taylor et al (2017).

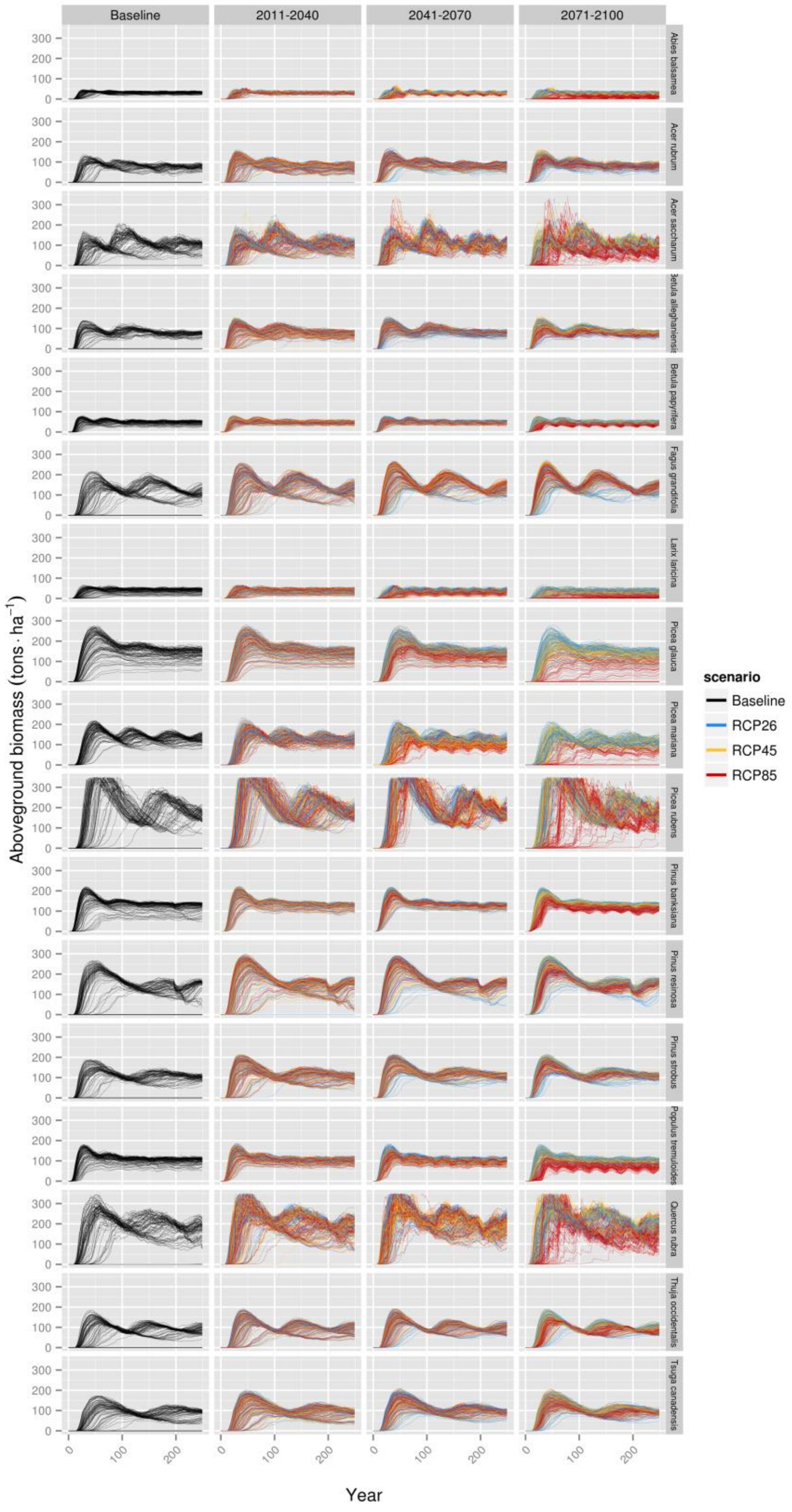

S2.1 Fig. Pure stand growth as simulated by PICUS.

The first set of parameters derived from PICUS outputs is maxAGB, defined as the maximum aboveground biomass that one species can attain on a given site ($g.m^{-2}$). It is analog to the carrying capacity of a given site, although a different value is attributed to each species. To set maxAGB for each combination of species, landtypes, climate scenarios and time periods from PICUS simulations, we averaged the total living aboveground biomass of simulated stands after they reached a plateau, i.e. when aboveground biomass tends to stabilize after the early growth phase and when the biomass peak that is usually observed right before the initial cohorts start dying off around the age of 100-150 years, is reached. Some species show a clear plateau (e.g., *Picea* spp.), while other present more ample oscillation (S2.1 Fig.). In LANDIS-II Biomass Succession, the carrying capacity of a given landtype, i.e. the maximum amount of total aboveground biomass a given cell can sustain, is determined by the maxAGB of the species showing the highest value on this particular landtype.

maxANPP is the maximum aboveground net primary productivity of aboveground biomass in $g.m^{-2}.yr^{-1}$. It can only be achieved under free growth conditions, i.e. in the total absence of inter- or intraspecific competition. To derive maxANPP from PICUS outputs, we summed 1) the annual increments in living aboveground biomass and 2) the annual amount of dead biomass produced, and then we extracted the maximum values for each combinations of species, landtypes, climate scenarios and time periods. These values were typically observed in the early stages of stand development (S2.1 Fig.).

In LANDIS-II Biomass Succession, **SEP** is defined as the probability of a given species' cohort to successfully establish on a given landtype during one time step, granted that seeds reach it and that light conditions are adequate. It can range from 0 to 1. Estimation of SEP is less straightforward than those of maxAGB and maxANPP and it involves some level of subjectivity. In this experiment, we considered the amount of time necessary for the initial cohort to reach 1.3 m in height. This is a subjective way of defining a cohort as being established, but since this cohort's growth is unsuppressed by any competition and is solely limited by climate and soil conditions, we believe it is in agreement with the model's definition of SEP. Then we translated this time interval (t) into a probability by considering it as the average result of a random process associated with a constant annual probability of $1/t$. We thus simulated the establishment of a cohort as a Bernouilli trial conducted every year during a time step. As the time step that we used was 5 years, we computed

the probability of having at least one successful trial in 5 consecutive ones based on the binomial distribution (S2.2 Fig.).

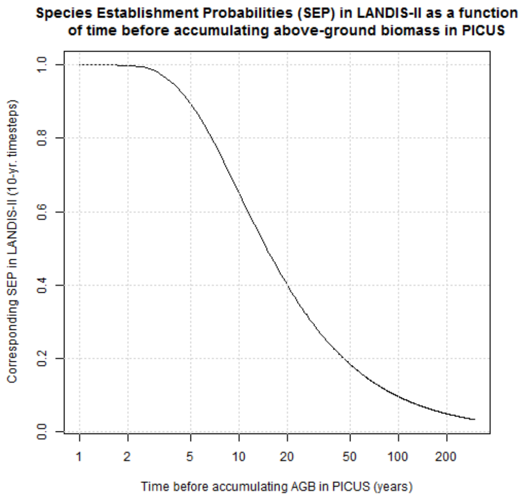

S2.2 Fig. Species Establishment Probabilities (*SEP*) in LANDIS-II as a function of time before accumulating aboveground biomass in PICUS

Distribution of the parameter values resulting from these computations is presented in S2.3 to S2.5 Figs.

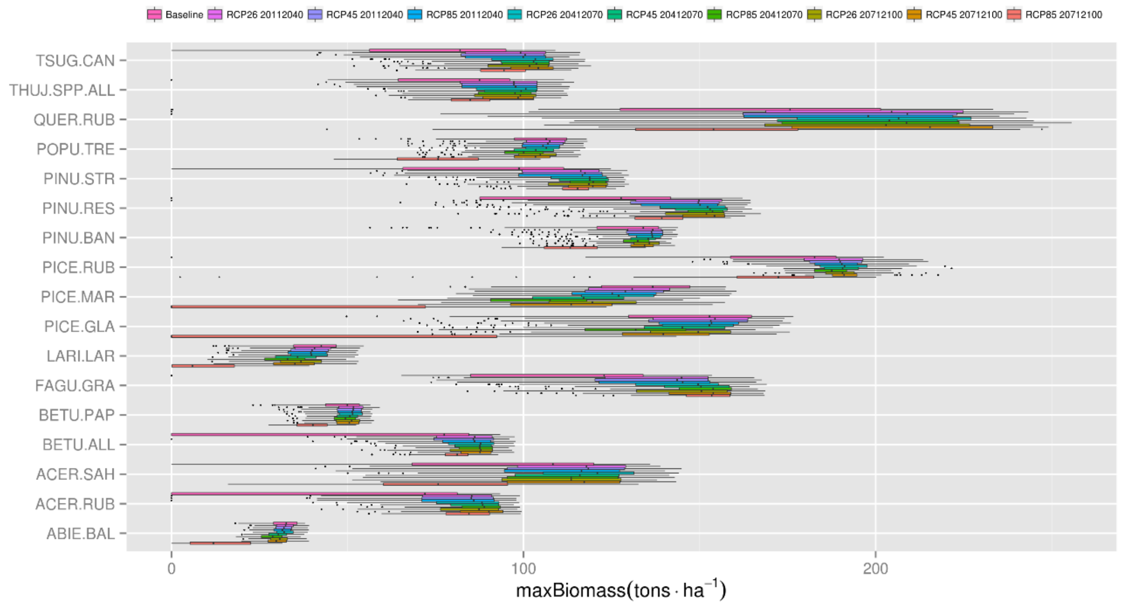

S2.3 Fig. Distribution of maxAGB among landtypes (N = 89, * based on uncorrected PICUS outputs).

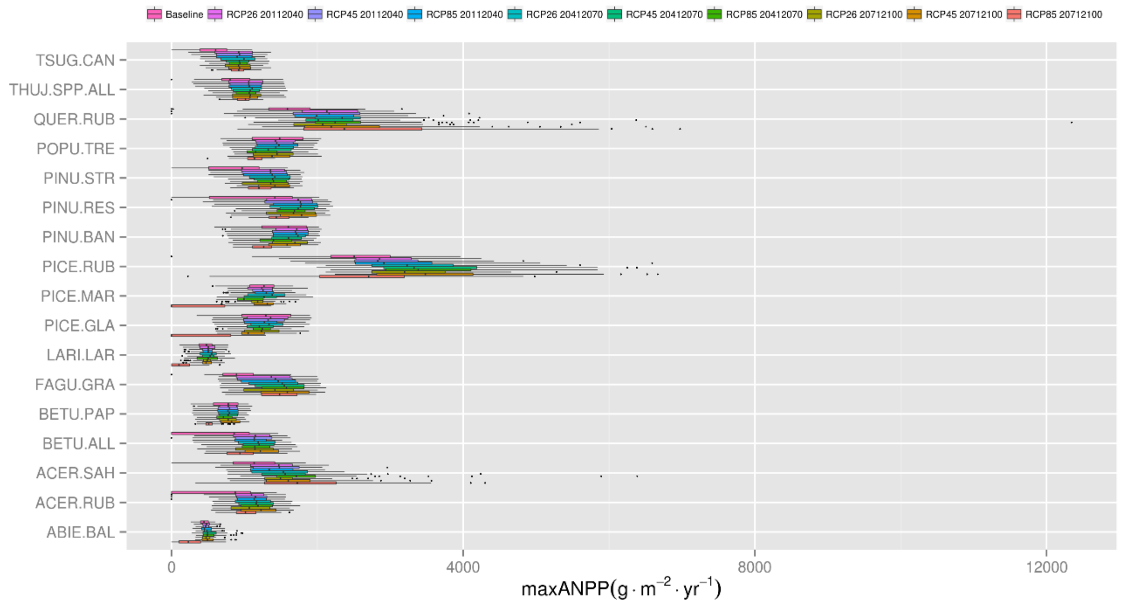

S2.4 Fig. Distribution of maxANPP among landtypes (N = 89, * based on uncorrected PICUS outputs).

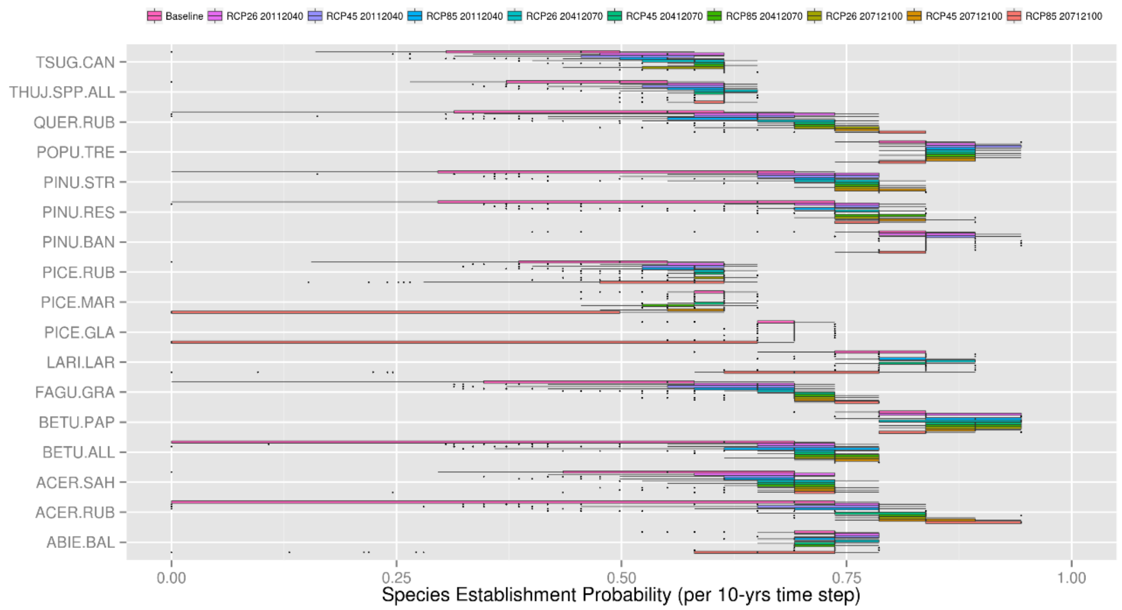

S2.5 Fig. Distribution of SEP among landtypes (N = 89, * based on uncorrected PICUS outputs).

Verification of cell-level (stand-level) emerging successional pathways

Context

To verify the successional pathways emerging from these parameters, we conducted cell-level simulations for a limited number of landtypes chosen among the 89 landtypes defined for that simulation area. They were subjectively selected among the most common landtypes to represent two contrasting, typical types of ecosystem.

At this stage, most of the parameters were already set, i.e. no further modifications were made to species life-history traits or dynamic inputs (apart from the bias correction presented in S3 Appendix). The purpose of these cell-level simulations was to set the last few parameters, namely the shade thresholds as well as the growth and mortality curves shape parameters. Verification was conducted by visualization of the emerging successional patterns. The trial-and-error processes that led to the final parameter values are described after the presentation of the cell-level simulation results.

In LANDIS-II Biomass Succession, the percentage of maximum aboveground biomass occupied throughout the simulation is used as a proxy for shade. Shade thresholds must be defined by the

user to create shade classes that may limit the establishment of species based on their shade tolerance. Shade tolerance and other important traits of all the species included in landscape-scale simulations are presented in Table 1 of the main article. As our simulations are currently parameterized, a given species' establishment becomes limited when a site's shade class reaches a value equal to the shade tolerance of that species. Establishment becomes impossible when the estimated shade class value exceeds the specific shade tolerance value.

Only a subset of the species used in LANDIS is included in the cell-level simulations presented above (S2.6-7 Figs.). The subset is based on species that are the most abundant in each landtype. Several other tests were conducted which led to the final species attribute parameters (Table 1).

Finally, we would like to stress that many of the parameters involved here are subject to imprecision and/or subjectivity. Moreover, they are shared among all simulated landtypes. A perfect parameterization is therefore virtually impossible to achieve.

Simulation setup

- A combination of five or six common species known to occur on this landtype start to grow from bare ground and interact for 1000 years with unlimited seed supply. The emerging successions are presented below;
- The cell size is 6.25 ha (250-m resolution);
- No disturbance occurs in those simulations;
- One cohort of each species is established at the beginning of each simulation. That is important to consider as it may differ from what mostly occurs in nature, where cohort establishment may be delayed for some species, especially after large-scale disturbances and when species are distributed in highly contagious manners;
- Because seed sources are not limited in those simulations, any locally extinct species can come back later in the simulations;
- Species- and landtype-specific parameters, maxAGB, maxANPP and SEP are derived from the stand-level model PICUS and were corrected for bias.

Simulated landtypes

Warm mesic mixedwoods (landtype 441_3)

The first simulated landtype is located in the southeastern portion of the simulated area, which consists of an incursion of temperate mixedwoods into an otherwise colder, boreal landscape (S2.6a Fig.) in the Lac-St-Jean region.

Boreal mesic softwoods (landtype 433_5)

The second simulated landtype is located in the northern and coldest portion of the simulated area. It is a typical of a mesic station in the Black spruce-Feathermoss bioclimatic domain (S2.6b Fig.).

Results regarding absolute and cumulative abundance, proportions, structural complexity and shade class are presented for both landtypes in S2.7-8 Figs.

a) b)

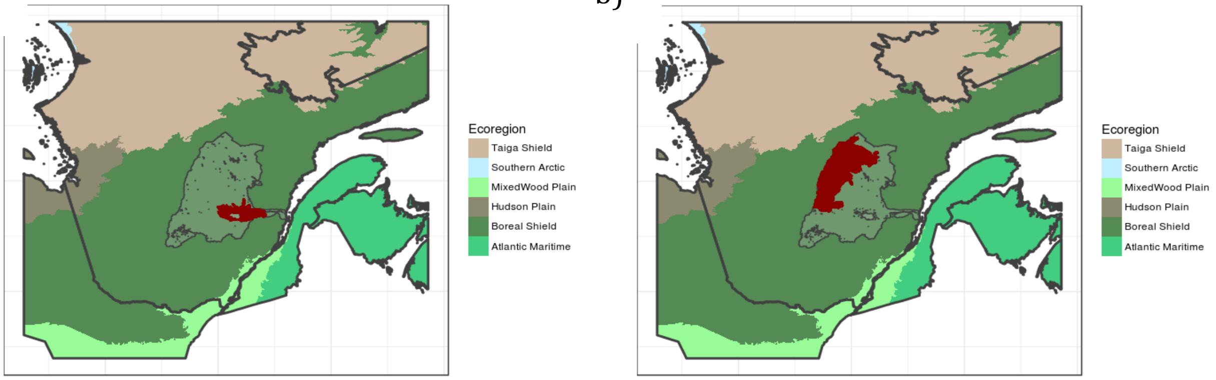

S2.6 Fig. Spatial distribution of a) temperate mesic mixedwoods (landtype 441_3), and b) boreal mesic softwoods (landtype 433_5).

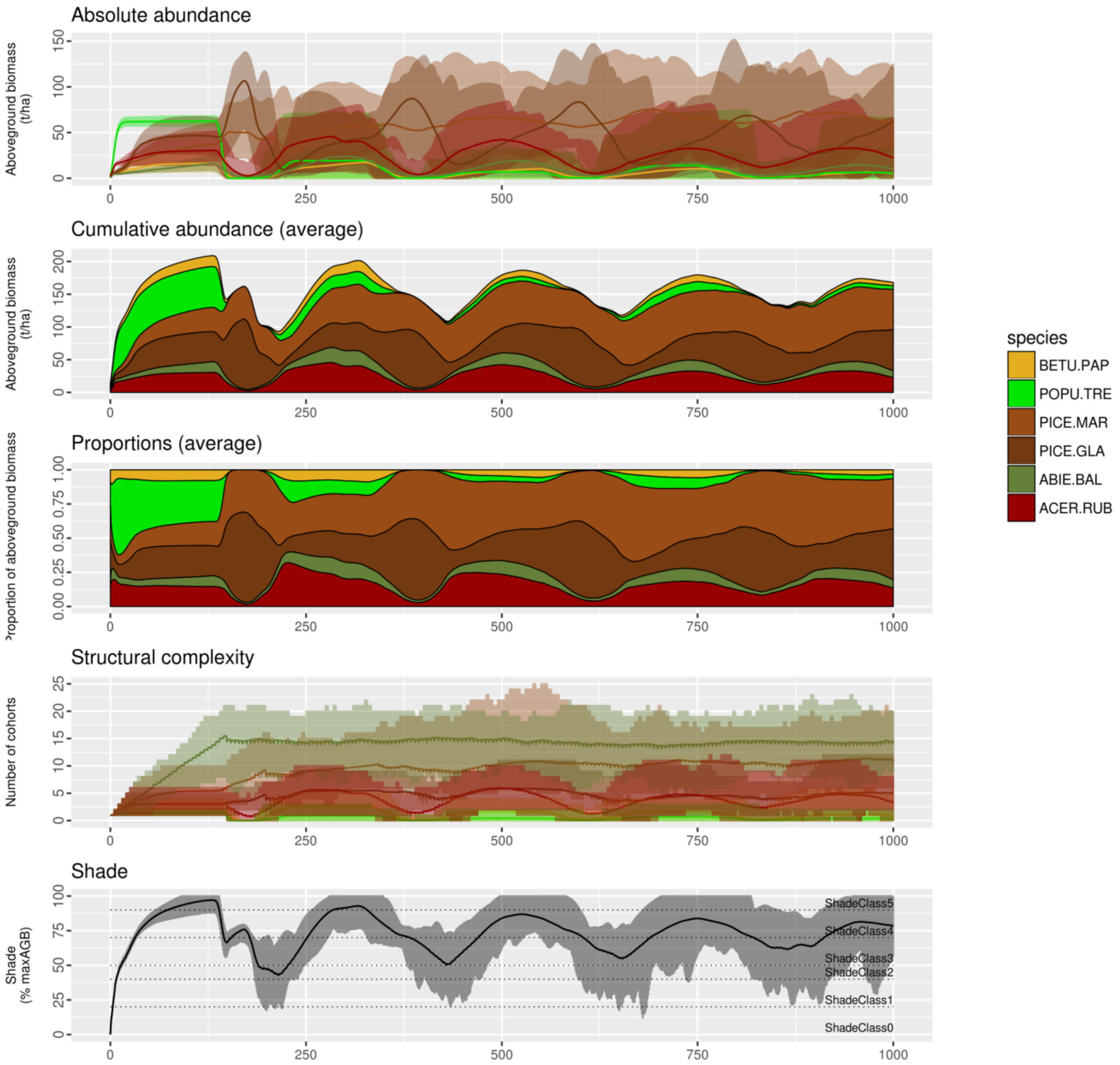

S2.7 Fig. Cell-level (stand-level) simulations for landtype 441_3 (temperate mesic mixedwoods; based on uncorrected PICUS outputs).

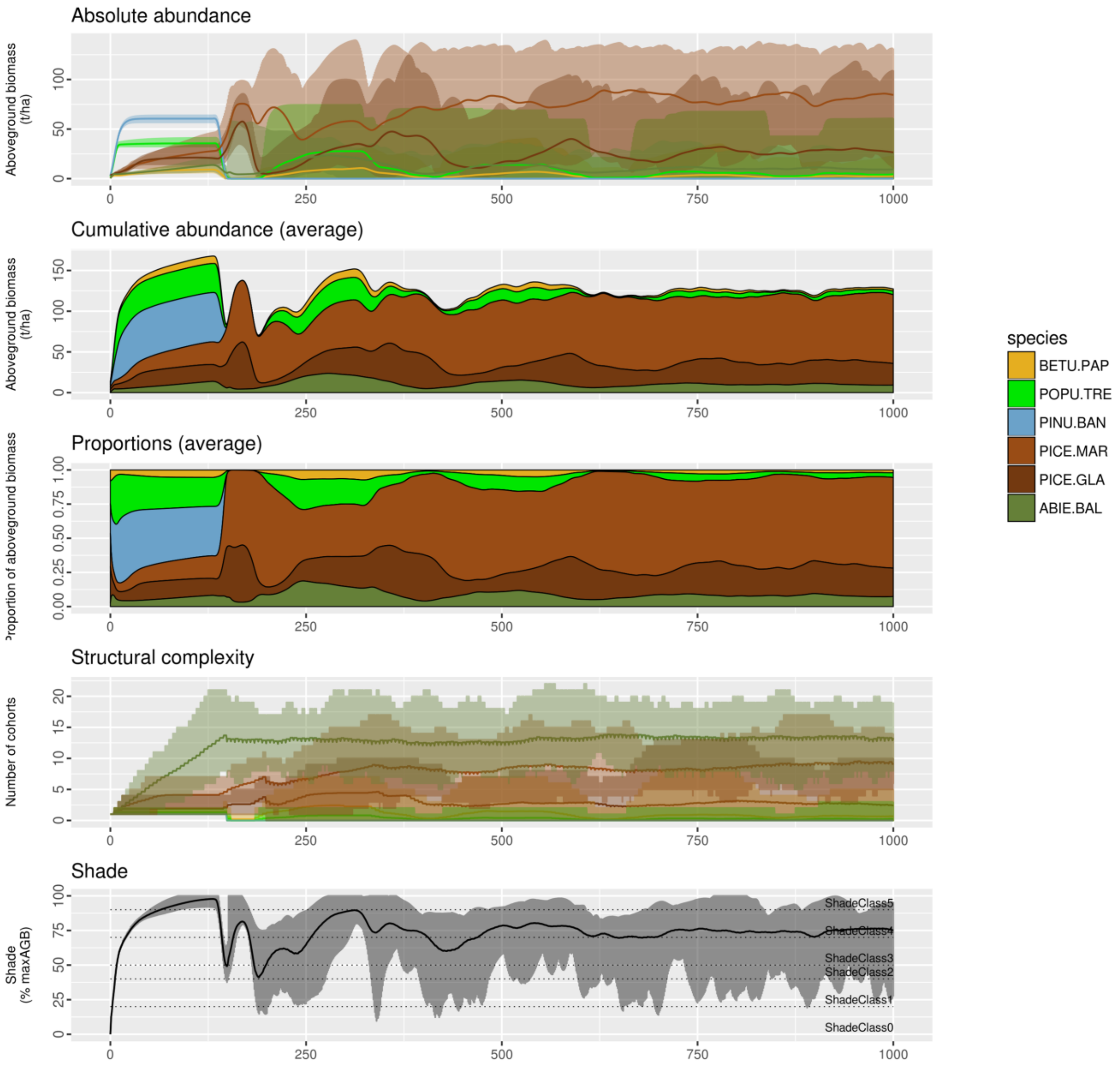

S2.8 Fig. Cell-level (stand-level) simulations for landtype 433_5 (boreal mesic softwoods; based on uncorrected PICUS outputs).

a) Verification of shade tolerance and shade classes

Shade tolerance is also a relatively well-documented aspect of the autoecology of each species, which, in Landis, affects the ability of a given species to establish itself under the canopy. We tried to represent the documented gradient of shade tolerance among species, although the limited resolution of this ordinal parameter (five levels) forced us to make some subjective choices. The

shade tolerance of each species was set prior to cell-level simulations and were not adjusted through a trial-and-error process. However, the thresholds that are used to define the shade classes based on the proportion of the available “growing space” (actual AGB / maximum AGB) can also be adjusted. To our knowledge, this is usually done through a trial-and-error process. Consequently, we tried to adjust these thresholds so that the timing of stand development, from a composition perspective, would roughly correspond to our best estimate of what is actually occurring in these landtypes.

b) Verification of mortality and growth curves shape parameters

Finally, we had to set two shape parameters: the **mortalityShape** and the **growthShape** parameters. Because they are subjective and abstract, they were set at the very end. The mortality shape parameter proved to be critical in determining how much of a given species’ cohorts would persist when approaching their maximum longevity, while the growth shape parameter helped determine in large part the extent and rate at which newly established cohorts could fill in the growing space made available after other cohorts were affected by mortality.

In order to simplify the process of manually setting these parameters for each species as well as to minimize the level of subjectivity involved, we lumped species into two groups based on their longevity (short-lived species: ≤ 150 years; long-lived species: > 150 years), to which the same mortalityShape and growthShape values were attributed. This is based on the observation that the growth of short-lived species generally takes off more rapidly than that of long-lived species, and that their mortality generally occurs in a more “condensed” portion of their lives, i.e. closer to the end of their maximum longevity, when compared with long-lived species.

The growth shape parameter was set to the maximum possible value for long-lived species (1) while short-lived species were attributed the smallest possible value (0). By making it so, the maximum growth potential of long-lived species was reached later in the simulation as opposed to the maximum growth potential of short-lived species, which was reached at a very young age. We found that this rule was simple enough to be justifiable using basic and easily described assumptions. More importantly, it allowed for a plausible level of species coexistence in undisturbed/steady-state stands, as well as a plausible increase in opportunistic species after partial disturbances (not shown here).

More information about all these parameters can be found in the LANDIS-II Biomass Succession Extension documentation (Scheller 2013) and in the original paper by Scheller and Mladenoff (2004).

References

Taylor AR, Boulanger Y, Price DT, Cyr D, McGarrigle E, Rammer W, Kershaw JA. Rapid 21st century climate change projected to shift composition and growth of Canada's Acadian Forest Region. For Ecol Manage 2017; 405: 284-294.
