# Supplementary material for: Harvesting interacts with climate change to affect future habitat quality of a focal species in eastern Canada’s boreal forest
Source: PLoS One. 2018 Feb 7;13(2):e0191645. doi: 10.1371/journal.pone.0191645 (PMC5802891; doi:10.1371/journal.pone.0191645)
Supplement: S3 Appendix — (PDF) [file pone.0191645.s003.pdf]

S3 Appendix

Bias correction of LANDIS-II Biomass Succession dynamic inputs

After the LANDIS-II Biomass Succession dynamics inputs were derived from the PICUS simulation (cf S2 Appendix), we tested for any important biases in the initial aboveground biomass (AGB) of one or many species as simulated during the LANDIS-II Biomass Succession spin-up phase. As a reference, we used the estimation of AGB from Beaudoin et al. (2014), also part of the National Forest Inventory (NFI; https://nfi.nfis.org/en/) (S3.1 Fig.).

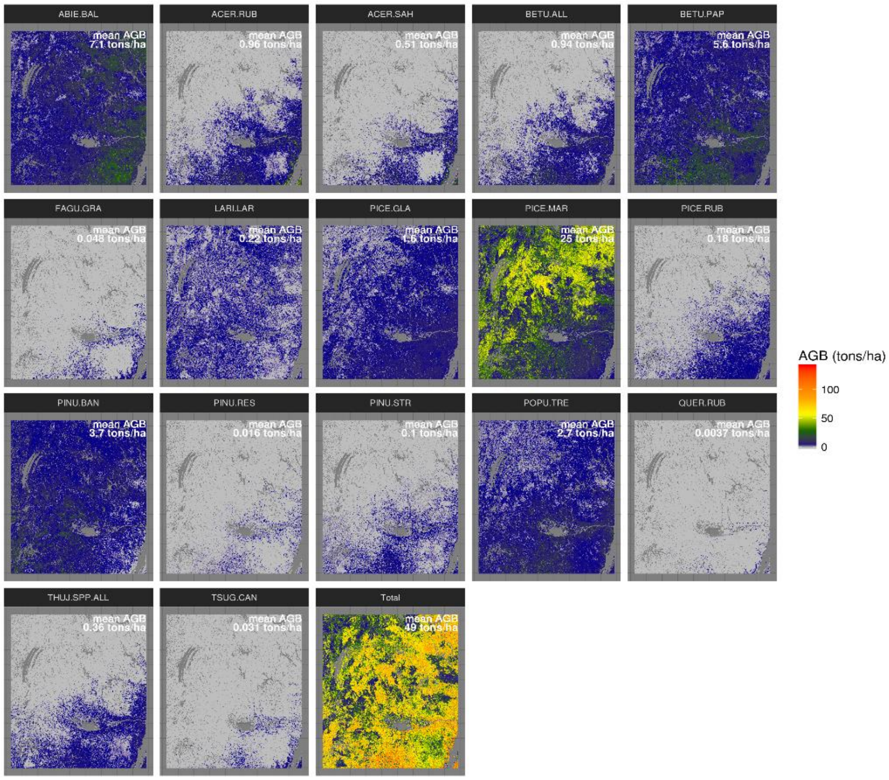

S3.1 Fig. Initial aboveground biomass of the most common tree species in the study area and surroundings, as estimated from Beaudoin et al. (2014) and reported in the Canadian National Forest Inventory (https://nfi.nfis.org/en/).

The differences between initial AGB as simulated during the LANDIS-II spin up phase and our reference (S3.2 Fig.) reveal that total initial AGB was overestimated by over 44 tons/ha on average, indicating considerable, mainly positive, biases in PICUS-derived dynamic inputs, namely maximum biomass (maxAGB) and/or maximum annual net primary productivity (maxANPP). Positive biases were observed for most of the most abundant species, mainly *Pinus banksiana* and *Picea mariana*, but also *Populus tremuloides* and other *Picea* spp. Negative biases were also observed but were less common. The only common species associated with a negative bias were *Abies balsamea* and *Betula papyrifera*. Our experience in other study areas and as well as the cell-level simulations conducted for this study area (S2 Appendix) suggested that the negative bias associated with *Abies balsamea* was problematic. Indeed, this late successional species could never achieve dominance and cast sufficient shade to suppress the establishment of less shade-tolerant species, even in its preferred landtypes. This was explained by an overemphasized sensitivity of that species to limited water availability in the current PICUS simulation set-up (Taylor et al. 2017).

Therefore, we deemed necessary to apply a bias correction that had two objectives: 1) to adjust the average initial total biomass within the landscape so that the one produced during the spin-up phase would correspond to the one reported in the NFI cover maps, mainly by multiplying all maxAGB by a constant scalar (maxANPP was kept proportional by multiplying it by the same value), and 2) to boost the relative importance of *Abies balsamea*, which turned out to be systematically underestimated, in a way that would enhance the realism of the simulations by further multiplying that species' maxAGB and maxANPP by another scalar. To achieve this correction, we conducted a two-step optimization through which we tried to find the best combination of these two scalars, in conjunction with the calibration of the SpinupMortalityFraction parameter, which adds background mortality during the biomass spin-up phase (Scheller and Mladenoff 2015) and therefore also influence initial AGB.

We conducted a series of simulations with a duration of 0 year (spin-up phase only) using the dynamic inputs derived from the PICUS simulations for the baseline climate scenario. In each one of these simulations, we varied three values following a full factorial design.

- We already knew that we had to decrease the initial AGB, so we multiplied the maxAGB and max ANPP of all species by values ranging from 0.4 to 1.
- We ran simulations with SpinupMortalityFraction ranging from 0 to 0.025.

- Finally, we multiplied the maxAGB and maxANPP of *Abies balsamea* by values that aimed to boost its mean maxAGB to values equivalent to 20 to 100% of the mean maxAGB of the species with the highest mean maxAGB. In the case of this landscape, it was *Picea rubens* (S2 Appendix).

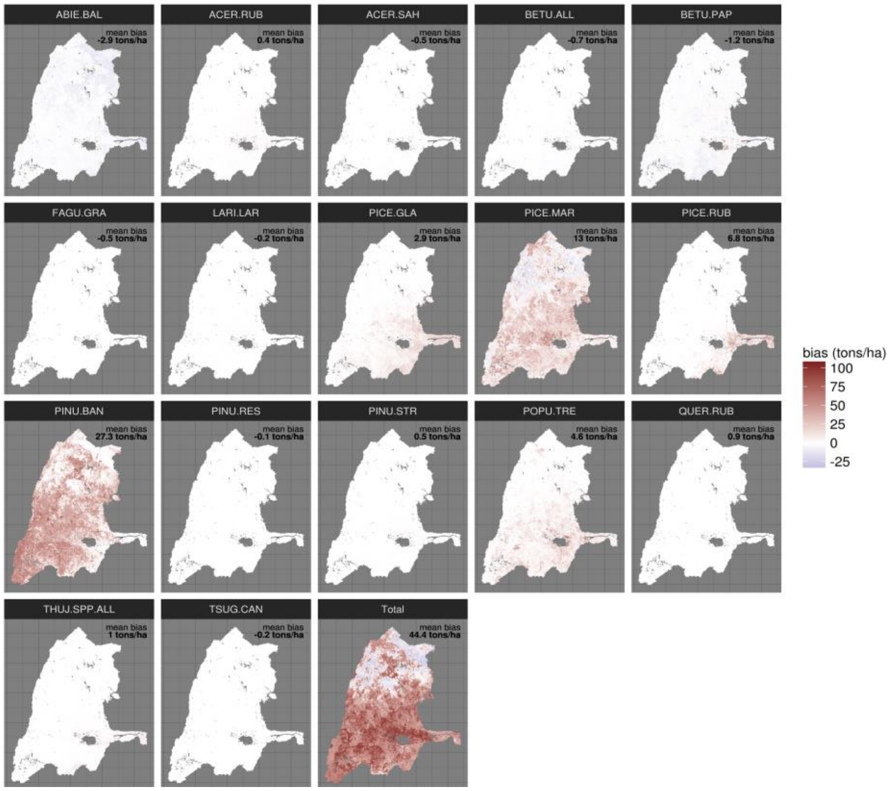

S3.2 Fig. Initial biases in aboveground biomass after the LANDIS-II Biomass Succession spin-up phase with no bias correction applied to PICUS-derived dynamic inputs. Mean biases only consider cells where the species is present (no zero values considered).

The first optimization step consisted in adjusting the average total AGB. Total AGB is sensitive to all of the above-mentioned values, but to a variable extent. Because it is most sensitive to the multiplier that is applied to all species, followed by the SpinupMortalityFraction, we first tried to find combinations of these two values that roughly corrected the average initial AGB. In the case of this landscape, a multiplier of 0.4 applied to all species' maxAGB (and maxANPP), combined with a SpinupMortalityFraction of 0.005, appeared to considerably improve the situation (S3.3a Fig.).

Then, using the maxAGB multiplier determined in the first step (0.4), we searched a two-dimensional space made of a range of SpinupMortalityFraction values and *A. balsamea*-specific multipliers to minimize the average dissimilarity between the community composition of each cell resulting from the spin-up phase and the community as described in the NFI. The dissimilarity index used was the Euclidian distance, which was computed from relative abundances. In the present case, a SpinupMortalityFraction of 0.005 was confirmed and a multiplier aimed at boosting the average maxAGB of *A. balsame*a to 30% of the average maxAGB of the species with the highest value (*P. rubens*) minimized the dissimilarity between the simulated communities and those described in the NFI (S3.3b Fig.).

The actual multiplier that was applied to the maxAGB values of *A. balsamea* for it to reach 30% of that of *P. Rubens* was 1.6774.

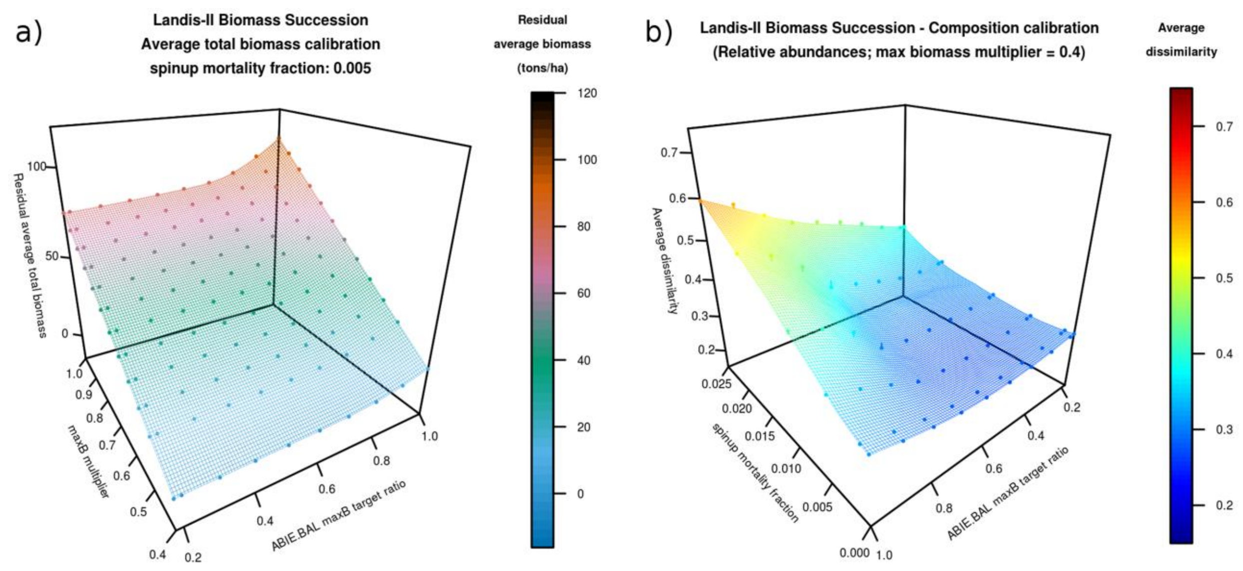

S.3.3 Fig. Calibration of LANDIS-II Biomass Succession dynamic inputs which (a) we adjusted the average total aboveground biomass by applying a correction factor to all species growth parameters, and (b) we adjusted the relative importance of *Abies balsamea* by boosting its own growth parameters to minimize the dissimilarity between simulated communities and

Then, because we assumed that all PICUS-derived parameters were affected by the same bias, we applied the same bias correction to all dynamic inputs, i.e. for all climate change scenarios.

The residual bias was considerably reduced by the bias correction. The average bias in total AGB after the bias correction was applied is -1.5 tons/ha (S3.4 Fig.), with the most common species

seeing their bias considerably reduced when compared with those obtained from the uncorrected dynamic inputs.

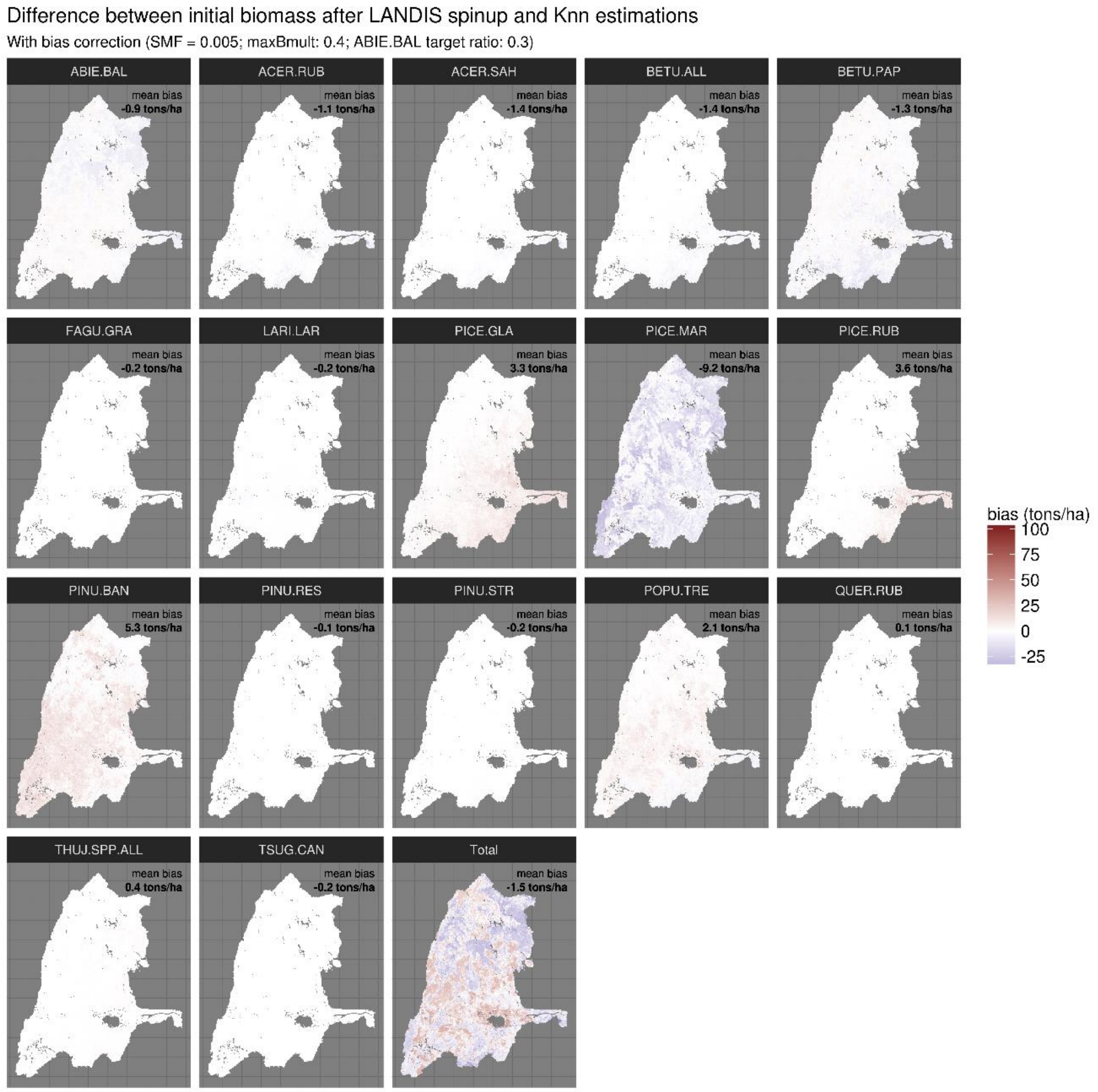

S3.4 Fig. Initial biases in aboveground biomass after the LANDIS-II Biomass Succession spin-up phase with a bias correction applied to PICUS-derived dynamic inputs. Mean biases only consider cells where species are present (no zero values considered).

References

Beaudoin A, Bernier PY, Guindon L, Villemaire P, Guo XJ, Stinson G, Bergeron T, Magnussen S, Hall RJ. Mapping attributes of Canada's forests at moderate resolution through kNN and MODIS imagery. Can J For Res 2014; 44: 521-532.

Scheller RM, Mladenoff DJ. A forest growth and biomass module for a landscape simulation model, LANDIS: design, validation, and application. Ecol Modell 2004; 180: 211–229.

Taylor AR, Boulanger Y, Price DT, Cyr D, McGarrigle E, Rammer W, Kershaw JA. Rapid 21st century climate change projected to shift composition and growth of Canada’s Acadian Forest Region. For Ecol Manage 2017; 405: 284-294.
