# Supplementary material for: Harvesting interacts with climate change to affect future habitat quality of a focal species in eastern Canada’s boreal forest
Source: PLoS One. 2018 Feb 7;13(2):e0191645. doi: 10.1371/journal.pone.0191645 (PMC5802891; doi:10.1371/journal.pone.0191645)
Supplement: S4 Appendix — (PDF) [file pone.0191645.s004.pdf]

S4 Appendix

Natural and anthropogenic disturbances as simulated in LANDIS-II

Fire and spruce budworm parameters used in LANDIS-II

1. **Base Fire input files**

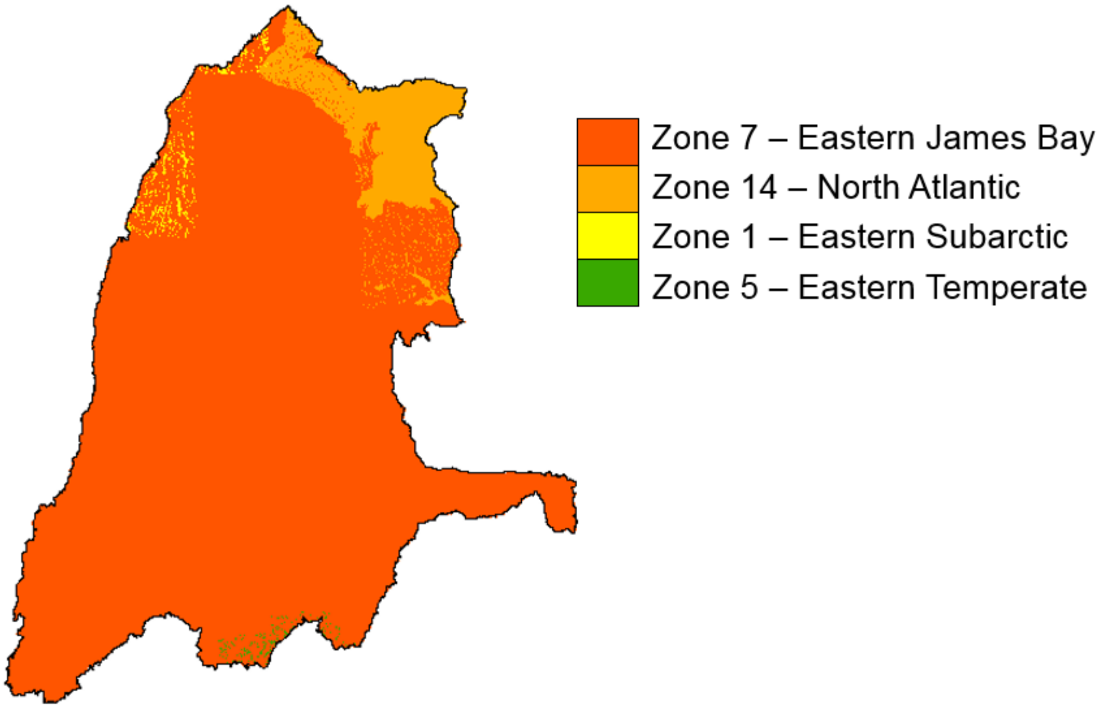

S4.1 Fig. Fire regions used by the Base Fire extension. Zone names referred to those used by Boulanger et al. (2014).

a. Baseline climate

```
LandisData "Base Fire"
Timestep  5
>> Fire Region Parameters
>> Fire
>> Region Map Mean Min Max Ignition
>> Name Code Size Size Size Prob k
>> ----------------------------------------------
ZONE_1_baseline_0 1 6484 200 70283 8.69926125e-07 1108
ZONE_5_baseline_0 5 2073 200 46230 8.638969375e-07 3500
ZONE_7_baseline_0 9 9861 200 263670 3.9367731875e-06 4
ZONE_14_baseline_0 13 6619 200 200040 3.510331625e-06 50
ZONE_1_baseline_10 17 6484 200 70283 8.69926125e-07 1108
ZONE_5_baseline_10 21 2073 200 46230 8.638969375e-07 3500
ZONE_7_baseline_10 25 9861 200 263670 3.9367731875e-06 4
ZONE_14_baseline_10 29 6619 200 200040 3.510331625e-06 50
ZONE_1_baseline_40 33 6484 200 70283 8.69926125e-07 1108
ZONE_5_baseline_40 37 2073 200 46230 8.638969375e-07 3500
ZONE_7_baseline_40 41 9861 200 263670 3.9367731875e-06 4
ZONE_14_baseline_40 45 6619 200 200040 3.510331625e-06 50
ZONE_1_baseline_70 49 6484 200 70283 8.69926125e-07 1108
ZONE_5_baseline_70 53 2073 200 46230 8.638969375e-07 3500
ZONE_7_baseline_70 57 9861 200 263670 3.9367731875e-06 4
ZONE_14_baseline_70 61 6619 200 200040 3.510331625e-06 50
InitialFireRegionsMap  "H:\YAN\LANDIS\CBFA\LSJ\fire-regions_LSJ_baseline_0.tif"
DynamicFireRegionTable << Optional
>>Year FileName
5 "H:\YAN\LANDIS\CBFA\LSJ\fire-regions_LSJ_baseline_0.tif"
15 "H:\YAN\LANDIS\CBFA\LSJ\fire-regions_LSJ_baseline_10.tif"
45 "H:\YAN\LANDIS\CBFA\LSJ\fire-regions_LSJ_baseline_40.tif"
75 "H:\YAN\LANDIS\CBFA\LSJ\fire-regions_LSJ_baseline_70.tif"
FuelCurveTable
>> Ecoregion S5 S4 S3 S2 S1
ZONE_1_baseline_0 -1 -1 -1 -1 10
ZONE_5_baseline_0 -1 -1 -1 -1 10
ZONE_7_baseline_0 -1 -1 -1 -1 10
ZONE_14_baseline_0 -1 -1 -1 -1 10
ZONE_1_baseline_10 -1 -1 -1 -1 10
ZONE_5_baseline_10 -1 -1 -1 -1 10
ZONE_7_baseline_10 -1 -1 -1 -1 10
ZONE_14_baseline_10 -1 -1 -1 -1 10
ZONE_1_baseline_40 -1 -1 -1 -1 10
ZONE_5_baseline_40 -1 -1 -1 -1 10
ZONE_7_baseline_40 -1 -1 -1 -1 10
ZONE_14_baseline_40 -1 -1 -1 -1 10
ZONE_1_baseline_70 -1 -1 -1 -1 10
ZONE_5_baseline_70 -1 -1 -1 -1 10
ZONE_7_baseline_70 -1 -1 -1 -1 10
ZONE_14_baseline_70 -1 -1 -1 -1 10
WindCurveTable
FireDamageTable
>> Cohort Age FireSeverity -
>> % of longevity FireTolerance
>> -------------- --------------
20% -2
50% -1
85% 0
100% 1
MapNames fire/severity_{timestep}.tif
LogFile fire/log.csv
SummaryLogFile fire/summary-log.csv
```

b. RCP 2.6

```
LandisData "Base Fire"
Timestep  5
>> Fire Region Parameters
>> Fire
>> Region Map Mean Min Max Ignition
>> Name Code Size Size Size Prob k
>> --------------------------------------------
ZONE_1_RCP26_0 2 6484 200 70283 8.69926125e-07 1108
ZONE_5_RCP26_0 6 2073 200 46230 8.638969375e-07 3500
ZONE_7_RCP26_0 10 9861 200 263670 3.9367731875e-06 4
ZONE_14_RCP26_0 14 6619 200 200040 3.510331625e-06 50
ZONE_1_RCP26_10 18 8986 200 70283 2.37388301738175e-06 1108
ZONE_5_RCP26_10 22 2123 200 46230 9.71093715330125e-07 3500
ZONE_7_RCP26_10 26 18239 200 263670 6e-06 1
ZONE_14_RCP26_10 30 6161 200 200040 2.392596100976e-06 75
ZONE_1_RCP26_40 34 9559 200 70283 2.93198640141712e-06 1108
ZONE_5_RCP26_40 38 2029 200 46230 1.17282219520506e-06 3500
ZONE_7_RCP26_40 42 20218 200 263670 7.2e-06 1
ZONE_14_RCP26_40 46 6232 200 200040 2.9672104461165e-06 75
ZONE_1_RCP26_70 50 9763 200 70283 3.24958116885619e-06 1108
ZONE_5_RCP26_70 54 2277 200 46230 1.11543464664269e-06 3500
ZONE_7_RCP26_70 58 20608 200 263670 8e-06 1
ZONE_14_RCP26_70 62 6089 200 200040 3.207817215338e-06 50
InitialFireRegionsMap  "H:\YAN\LANDIS\CBFA\LSJ\fire-regions_LSJ_RCP26_0.tif"
DynamicFireRegionTable << Optional
>>Year FileName
5 "H:\YAN\LANDIS\CBFA\LSJ\fire-regions_LSJ_RCP26_0.tif"
15 "H:\YAN\LANDIS\CBFA\LSJ\fire-regions_LSJ_RCP26_10.tif"
45 "H:\YAN\LANDIS\CBFA\LSJ\fire-regions_LSJ_RCP26_40.tif"
75 "H:\YAN\LANDIS\CBFA\LSJ\fire-regions_LSJ_RCP26_70.tif"
FuelCurveTable
>> Ecoregion S5 S4 S3 S2 S1
ZONE_1_RCP26_0 -1 -1 -1 -1 10
ZONE_5_RCP26_0 -1 -1 -1 -1 10
ZONE_7_RCP26_0 -1 -1 -1 -1 10
ZONE_14_RCP26_0 -1 -1 -1 -1 10
ZONE_1_RCP26_10 -1 -1 -1 -1 10
ZONE_5_RCP26_10 -1 -1 -1 -1 10
ZONE_7_RCP26_10 -1 -1 -1 -1 10
ZONE_14_RCP26_10 -1 -1 -1 -1 10
ZONE_1_RCP26_40 -1 -1 -1 -1 10
ZONE_5_RCP26_40 -1 -1 -1 -1 10
ZONE_7_RCP26_40 -1 -1 -1 -1 10
ZONE_14_RCP26_40 -1 -1 -1 -1 10
ZONE_1_RCP26_70 -1 -1 -1 -1 10
ZONE_5_RCP26_70 -1 -1 -1 -1 10
ZONE_7_RCP26_70 -1 -1 -1 -1 10
ZONE_14_RCP26_70 -1 -1 -1 -1 10
WindCurveTable
FireDamageTable
>> Cohort Age FireSeverity -
>> % of longevity FireTolerance
>> -------------- --------------
20% -2
50% -1
85% 0
100% 1
MapNames fire/severity_{timestep}.tif
LogFile fire/log.csv
SummaryLogFile fire/summary-log.csv
```

c. RCP 4.5

```
LandisData "Base Fire"
Timestep  5
>> Fire Region Parameters
>> Fire
>> Region Map Mean Min Max Ignition
>> Name Code Size Size Size Prob k
>> --------------------------------------------
ZONE_1_RCP45_0 3 6484 200 70283 8.69926125e-07 1108
ZONE_5_RCP45_0 7 2073 200 46230 8.638969375e-07 3500
ZONE_7_RCP45_0 11 9861 200 263670 3.9367731875e-06 4
ZONE_14_RCP45_0 15 6619 200 200040 3.510331625e-06 50
ZONE_1_RCP45_10 19 8786 200 70283 2.40337439894962e-06 1108
ZONE_5_RCP45_10 23 2344 200 46230 1.27887777497875e-06 3500
ZONE_7_RCP45_10 27 17540 200 263670 7.2e-06 1
ZONE_14_RCP45_10 31 5910 200 200040 2.85045927150169e-06 50
ZONE_1_RCP45_40 35 9966 200 70283 3.96921263526938e-06 1108
ZONE_5_RCP45_40 39 2411 200 46230 1.58261580236606e-06 3500
ZONE_7_RCP45_40 43 20288 200 263670 1.119e-05 1
ZONE_14_RCP45_40 47 5897 200 200040 4.68983032687219e-06 50
ZONE_1_RCP45_70 51 10106 200 70283 4.17850174821863e-06 1108
ZONE_5_RCP45_70 55 2528 200 46230 1.87157786460469e-06 3500
ZONE_7_RCP45_70 59 21281 200 263670 1.4e-05 1
ZONE_14_RCP45_70 63 5985 200 200040 4.879665475372e-06 50
InitialFireRegionsMap  "H:\YAN\LANDIS\CBFA\LSJ\fire-regions_LSJ_RCP45_0.tif"
DynamicFireRegionTable << Optional
>>Year FileName
5 "H:\YAN\LANDIS\CBFA\LSJ\fire-regions_LSJ_RCP45_0.tif"
15 "H:\YAN\LANDIS\CBFA\LSJ\fire-regions_LSJ_RCP45_10.tif"
45 "H:\YAN\LANDIS\CBFA\LSJ\fire-regions_LSJ_RCP45_40.tif"
75 "H:\YAN\LANDIS\CBFA\LSJ\fire-regions_LSJ_RCP45_70.tif"
FuelCurveTable
>> Ecoregion S5 S4 S3 S2 S1
ZONE_1_RCP45_0 -1 -1 -1 -1 10
ZONE_5_RCP45_0 -1 -1 -1 -1 10
ZONE_7_RCP45_0 -1 -1 -1 -1 10
ZONE_14_RCP45_0 -1 -1 -1 -1 10
ZONE_1_RCP45_10 -1 -1 -1 -1 10
ZONE_5_RCP45_10 -1 -1 -1 -1 10
ZONE_7_RCP45_10 -1 -1 -1 -1 10
ZONE_14_RCP45_10 -1 -1 -1 -1 10
ZONE_1_RCP45_40 -1 -1 -1 -1 10
ZONE_5_RCP45_40 -1 -1 -1 -1 10
ZONE_7_RCP45_40 -1 -1 -1 -1 10
ZONE_14_RCP45_40 -1 -1 -1 -1 10
ZONE_1_RCP45_70 -1 -1 -1 -1 10
ZONE_5_RCP45_70 -1 -1 -1 -1 10
ZONE_7_RCP45_70 -1 -1 -1 -1 10
ZONE_14_RCP45_70 -1 -1 -1 -1 10
WindCurveTable
FireDamageTable
>> Cohort Age FireSeverity -
>> % of longevity FireTolerance
>> -------------- --------------
20% -2
50% -1
85% 0
100% 1
MapNames fire/severity_{timestep}.tif
LogFile fire/log.csv
SummaryLogFile fire/summary-log.csv
```

d. RCP 8.5

```
LandisData "Base Fire"
Timestep  5
>> Fire Region Parameters
>> Fire
>> Region Map Mean Min Max Ignition
>> Name Code Size Size Size Prob k
>> ------------------------------------------------
ZONE_1_RCP85_0 4 6484 200 70283 8.69926125e-07 1108
ZONE_5_RCP85_0 8 2073 200 46230 8.638969375e-07 3500
ZONE_7_RCP85_0 12 9861 200 263670 3.9367731875e-06 4
ZONE_14_RCP85_0 16 6619 200 200040 3.510331625e-06 50
ZONE_1_RCP85_10 20 9196 200 70283 2.65481253903969e-06 1108
ZONE_5_RCP85_10 24 2066 200 46230 9.93742025201625e-07 3500
ZONE_7_RCP85_10 28 18941 200 263670 7e-06 1
ZONE_14_RCP85_10 32 5806 200 200040 2.69800215045312e-06 50
ZONE_1_RCP85_40 36 10240 200 70283 4.73125023728606e-06 1108
ZONE_5_RCP85_40 40 2586 200 46230 1.68073370661419e-06 3500
ZONE_7_RCP85_40 44 19882 200 263670 1.119e-05 1
ZONE_14_RCP85_40 48 5947 200 200040 5.36190818828331e-06 50
ZONE_1_RCP85_70 52 10170 200 70283 6.75352465513894e-06 1108
ZONE_5_RCP85_70 56 2763 200 46230 2.72501093191694e-06 3500
ZONE_7_RCP85_70 60 17928 200 263670 2e-05 1
ZONE_14_RCP85_70 64 6520 200 200040 9.98539650074725e-06 50
InitialFireRegionsMap  "H:\YAN\LANDIS\CBFA\LSJ\fire-regions_LSJ_RCP85_0.tif"
DynamicFireRegionTable << Optional
>>Year FileName
5 "H:\YAN\LANDIS\CBFA\LSJ\fire-regions_LSJ_RCP85_0.tif"
15 "H:\YAN\LANDIS\CBFA\LSJ\fire-regions_LSJ_RCP85_10.tif"
45 "H:\YAN\LANDIS\CBFA\LSJ\fire-regions_LSJ_RCP85_40.tif"
75 "H:\YAN\LANDIS\CBFA\LSJ\fire-regions_LSJ_RCP85_70.tif"
FuelCurveTable
>> Ecoregion S5 S4 S3 S2 S1
ZONE_1_RCP85_0 -1 -1 -1 -1 10
ZONE_5_RCP85_0 -1 -1 -1 -1 10
ZONE_7_RCP85_0 -1 -1 -1 -1 10
ZONE_14_RCP85_0 -1 -1 -1 -1 10
ZONE_1_RCP85_10 -1 -1 -1 -1 10
ZONE_5_RCP85_10 -1 -1 -1 -1 10
ZONE_7_RCP85_10 -1 -1 -1 -1 10
ZONE_14_RCP85_10 -1 -1 -1 -1 10
ZONE_1_RCP85_40 -1 -1 -1 -1 10
ZONE_5_RCP85_40 -1 -1 -1 -1 10
ZONE_7_RCP85_40 -1 -1 -1 -1 10
ZONE_14_RCP85_40 -1 -1 -1 -1 10
ZONE_1_RCP85_70 -1 -1 -1 -1 10
ZONE_5_RCP85_70 -1 -1 -1 -1 10
ZONE_7_RCP85_70 -1 -1 -1 -1 10
ZONE_14_RCP85_70 -1 -1 -1 -1 10
WindCurveTable
FireDamageTable
>> Cohort Age FireSeverity -
>> % of longevity FireTolerance
>> -------------- --------------
20% -2
50% -1
85% 0
100% 1
MapNames fire/severity_{timestep}.tif
LogFile fire/log.csv
SummaryLogFile fire/summary-log.csv
```

2. Budworm BDA Input file

```
BDAAgentName          budworm
BDPCalibrator         1              << integer, parameter "a" in the BDP equation (#2 in user
Guide)
SRDMode               mean           << max or mean, SRD score based on the mean or max SRD

>>-------- Regional Outbreak Inputs -------------
TimeSinceLastEpidemic 20             << years
TemporalType          pulse  << pulse, variablepulse, or continuous
RandomFunction        RFuniform      << RFnormal or RFuniform, function used to determine
interval between outbreaks
RandomParameter1      32             << avg time interval between obks; Robert et al.2012 if
RFnormal, or max interval if RFuniform
RandomParameter2      32             << std dev. of interval between obks; Robert et al.2012 if
RFnormal, or min interval if RFuniform
MinROS                0              << regional obk severity; min. severity (without regards to
forest susceptibility or vulnerability)
MaxROS                3              << maximum potential severity

>>-------- Dispersal Inputs ----------------------
Dispersal             no             << yes or no
DispersalRate         50000          << ½ de 50km/year - from Royama 1984, it's the min. avg SBW
moths dispersal dist.(50-100km)
EpidemicThresh        0.5 << minimum BDP required to start an epicenter | originally=0.5
InitialEpicenterNum   5 << originally of 0
OutbreakEpicenterCoeff 0.01          << "The number of new epicenters (within outbreak zone)
                                     << will decrease with increasing c"
SeedEpicenter         yes            << yes or no
SeedEpicenterCoeff    10             << param. "c",eq.3(user guide);# of new epicenters,
decreases with increasing c; originally=0.01
DispersalTemplate     MaxRadius      << MaxRadius, 4N, 8N, 12N, or 24N; 8N=all cells (one-cell
radius), 24N=two-cell radius
>>InitialCondition    none           << map or none

>>-------- Neighborhood Resource Inputs -----------
NeighborFlag   yes                   << yes or no (determines whether NRD is used in calculating
BDP)
NeighborSpeedUp       none                  << none, 2x, 3x, or 4x (will use every cell in
neighborhood)
NeighborRadius 1000                  << meters
NeighborShape  uniform               << uniform, linear, or gaussian; weight of neighborhood
cells vs. distance to site
NeighborWeight 0.5                   <<  0.01 - 100; parameter "NW" in eq.2; 0.01:NRD weight=0%
of SRD,1:50%,10:91%,100=100% of SRD;

>>-------- Budworm Model Inputs (Budworm BDA additional section) -----------
>> SV values are compared to BDP to assign the obk an intensity class, then VulnProb takes over
to assign mortality prob.
>> This is where you determine how severely impacted the forest will be given a ROS=3, requires
calibration!
Class2_SV 0.33        << BDP value threshold to reach severity class 2
Class3_SV 0.67               << BDP value threshold to reach severity class 3
BFAgeCutoff  70       << age over which all fir cohorts are killed, below that, VulnProb takes
over; 0 to kill all cohorts

>>EcoregionModifiers
>>Ecoregion    Modifier; -1 to 1; see eq.1; -1 reduces SRD by 100%, 1 increases SRD by 100%;
constant through simulation


DisturbanceModifiers
>>Type  Duration      Value;  -1 to 1; impact decreases with time since disturbance
>>Wind  0             0.00
Fire   0              0.00

BDASpeciesParameters         << SRD=probability of defoliation, VRD=probability of mortality
from defoliation.
```

```
>> SRD values determine intensity of defoliation (BDP) through SV, whereas intensity class(vuln.) determined from the relative
>> scale of SV thresholds -
>>                                                   | Susceptibility
                              |
>>Species  MinorHost   2ndHost        MajorHost      | Class3                      Class2
       Class1          |      CFS
>>Name     Age SRDProb Age    SRDProb Age     SRDProb | Age   VulnProb       Age     VulnProb
       Age     VulnProb |     Conifer?
>>adapted BDA values
ABIE.BAL 0 0.25 20 0.5 40 1 0 0 20 0.42 50 0.85 yes
PICE.GLA 0 0.18 20 0.36 40 0.72 0 0 20 0.15 50 0.42 yes
PICE.RUB 0 0.1 20 0.2 40 0.41 20 0.13 50 0.36 999 1 yes
PICE.MAR 0 0.07 20 0.14 40 0.28 20 0.13 50 0.36 999 1 yes
>>IgnoredSpecies
>>ACER.RUB
>>ACER.SAH
>>BETU.ALL
>>BETU.PAP
>>FAGU.GRA
>>LARI.LAR
>>PINU.BAN
>>PINU.RES
>>PINU.STR
>>POPU.TRE
>>QUER.RUB
>>THUJ.SPP.ALL
>>TSUG.CAN
```

3. Projection of disturbance regimes

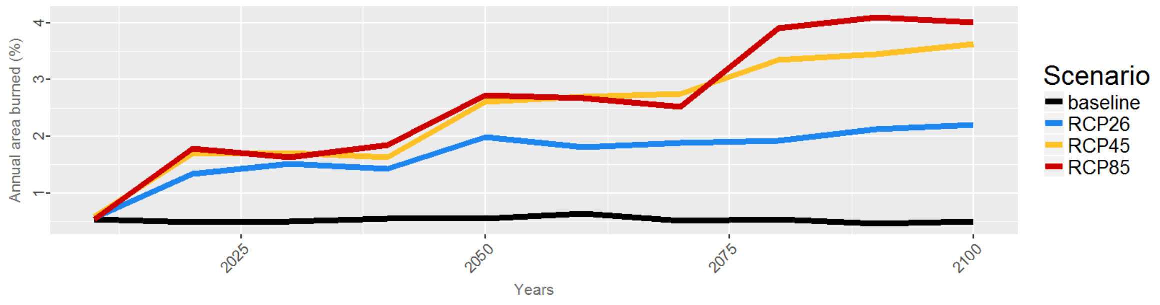

S4.2 Fig. Baseline and future annual area burned as estimated from models developed by Boulanger et al. (2014) and further updated for RCP scenarios (Gauthier et al. 2015). Values were kept constant after 2100. Values at year 2000 are those estimated for baseline climate.

a)

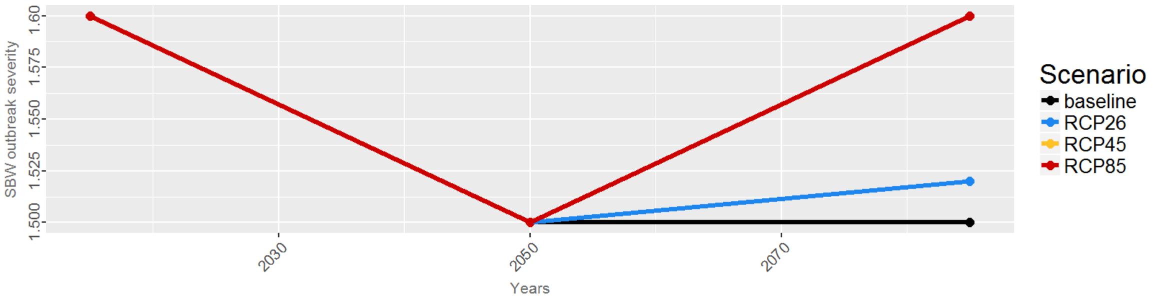

b)

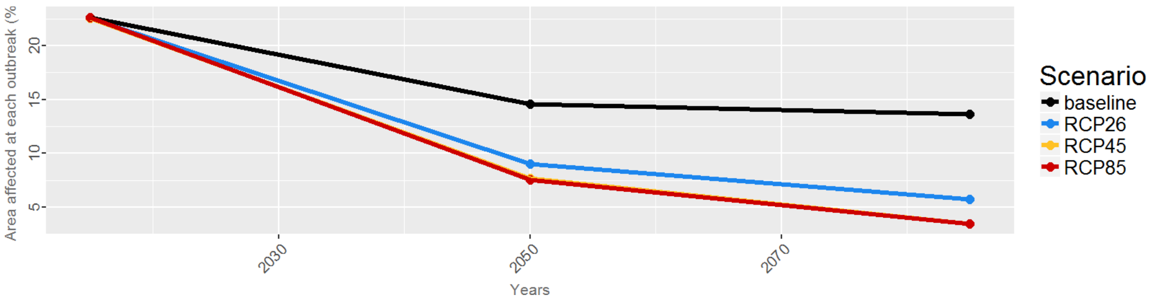

S4.3 Fig. a) Mean outbreak severity (on a 0 – 3 scale, 3 being the most severe) and b) total area affected by the spruce budworm during each outbreak within the study area under each of the four climate forcing scenarios.

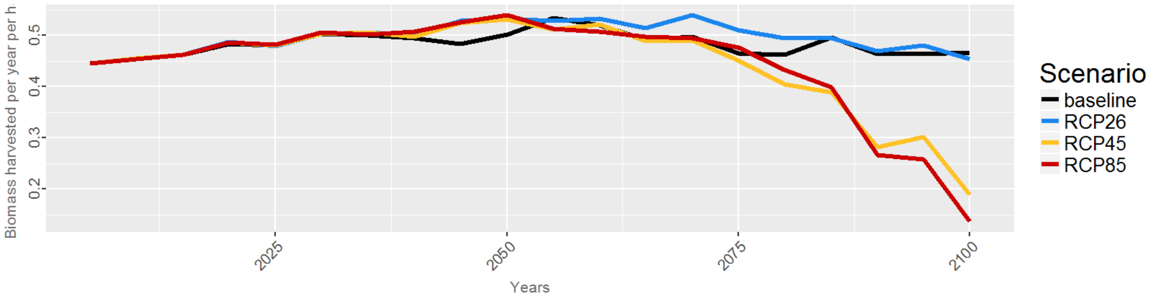

S4.4 Fig. Total biomass harvested in the study area under each of the four climate forcing scenarios

References

Boulanger Y, Gauthier S, Burton PJ. A refinement of models projecting future Canadian fire regimes using homogeneous fire regime zones. Can J For Res 2014; 44: 365–376.

Gauthier S, Bernier PY, Boulanger Y, Guo J, Guindon L, Beaudoin A, Boucher D. Vulnerability of timber supply to projected changes in fire regime in Canada's managed forests. Can J Forest Res 2015; 45: 1439-1447.
