# Supplementary material for: Harvesting interacts with climate change to affect future habitat quality of a focal species in eastern Canada’s boreal forest
Source: PLoS One. 2018 Feb 7;13(2):e0191645. doi: 10.1371/journal.pone.0191645 (PMC5802891; doi:10.1371/journal.pone.0191645)
Supplement: S5 Appendix — (PDF) [file pone.0191645.s005.pdf]

S5 Appendix

Trends in species-specific mean biomass differences (ΔB)

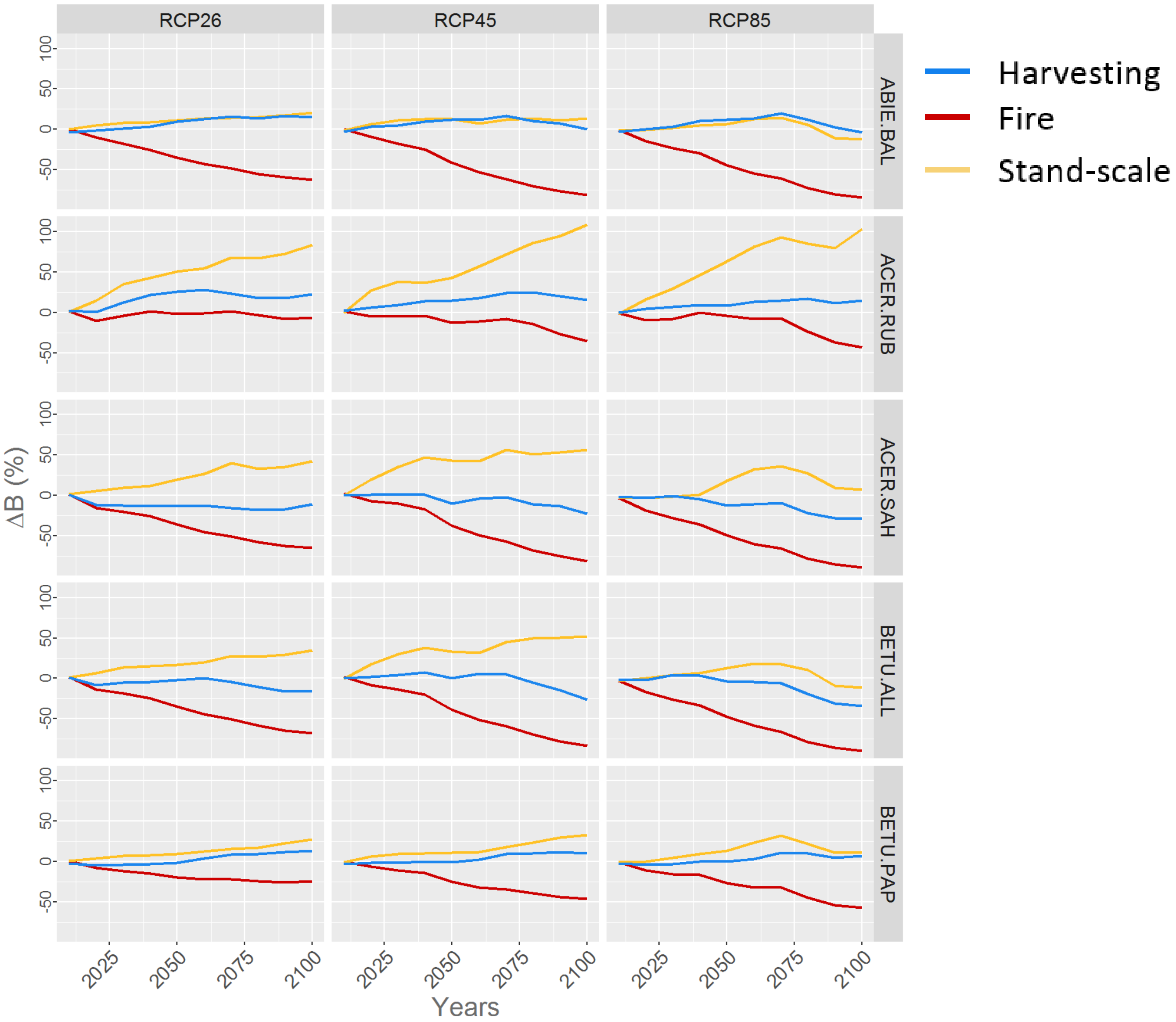

(continued…)

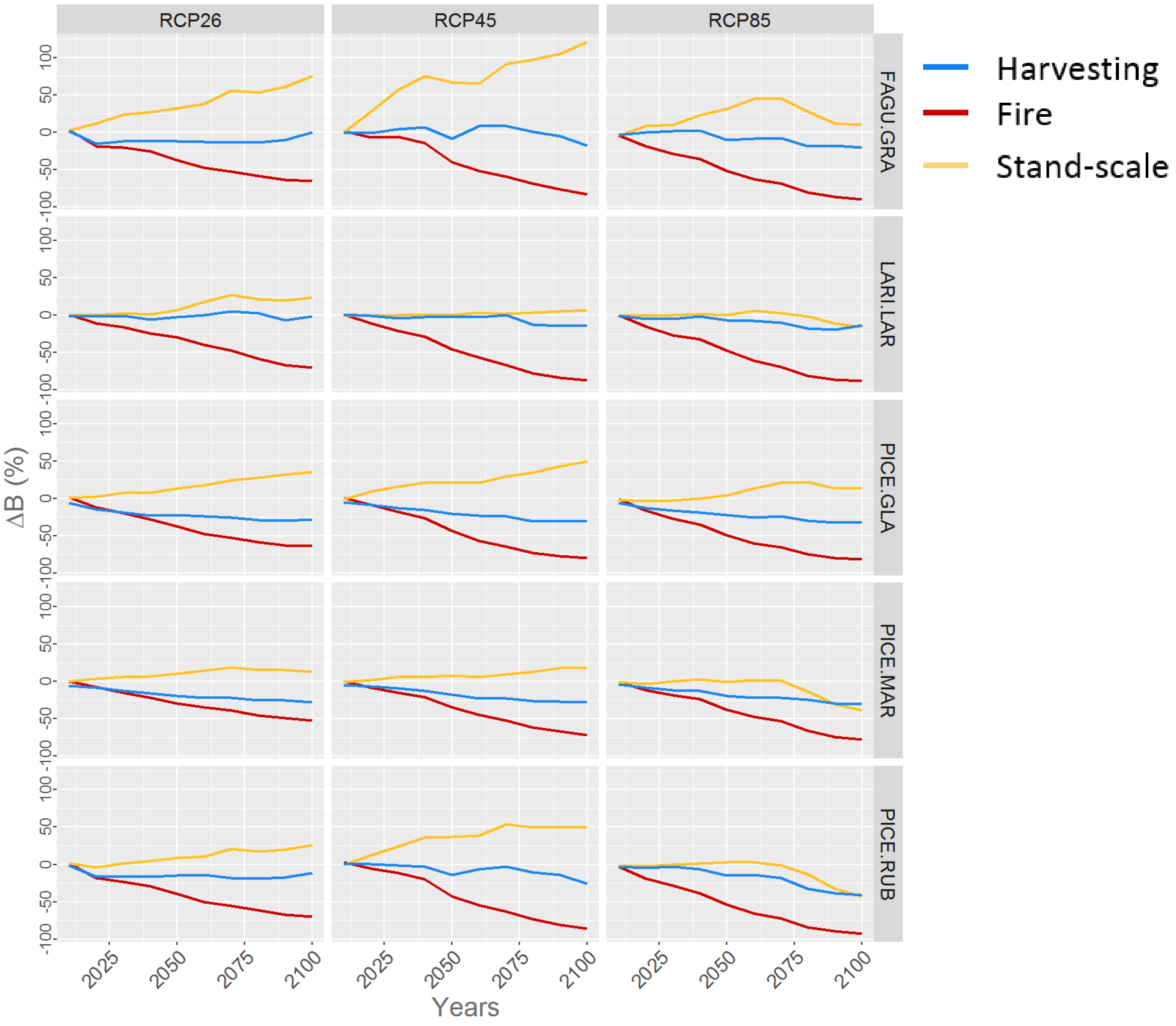

RCP26
RCP45
RCP85
FAGU.GRA
LARI.LAR
PICE.GLA
PICE.MAR
PICE.RUB
Harvesting
Fire
Stand-scale
ΔB (%)
Years
-100
-50
0
50
100
2025
2050
2075
2100

(continued…)

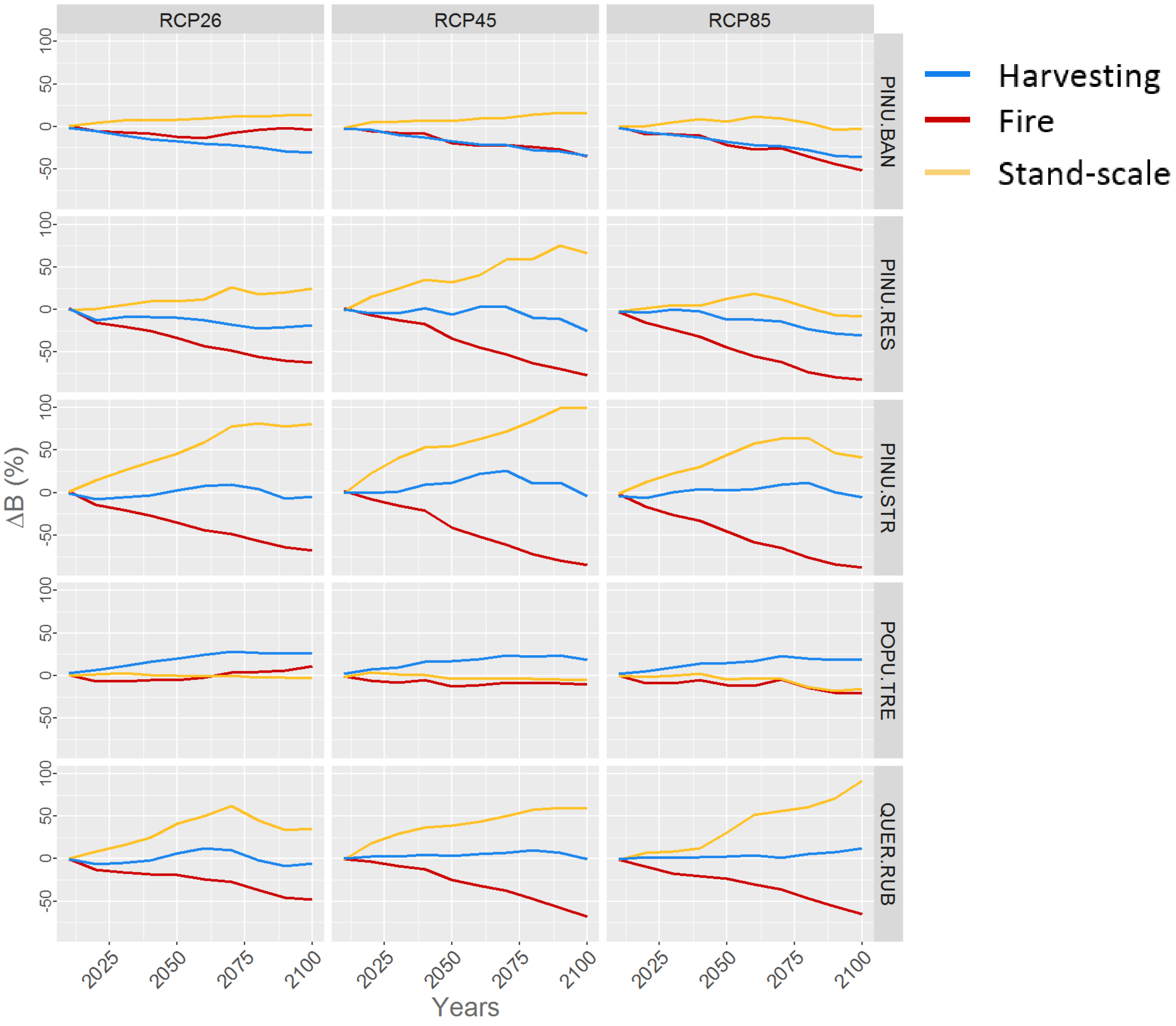

RCP26
RCP45
RCP85
PINU.BAN
PINU.RES
PINU.STR
POPU.TRE
QUER.RUB
ΔB (%)
100
50
0
-50
2025
2050
2075
2100
Years
Harvesting
Fire
Stand-scale

(continued…)

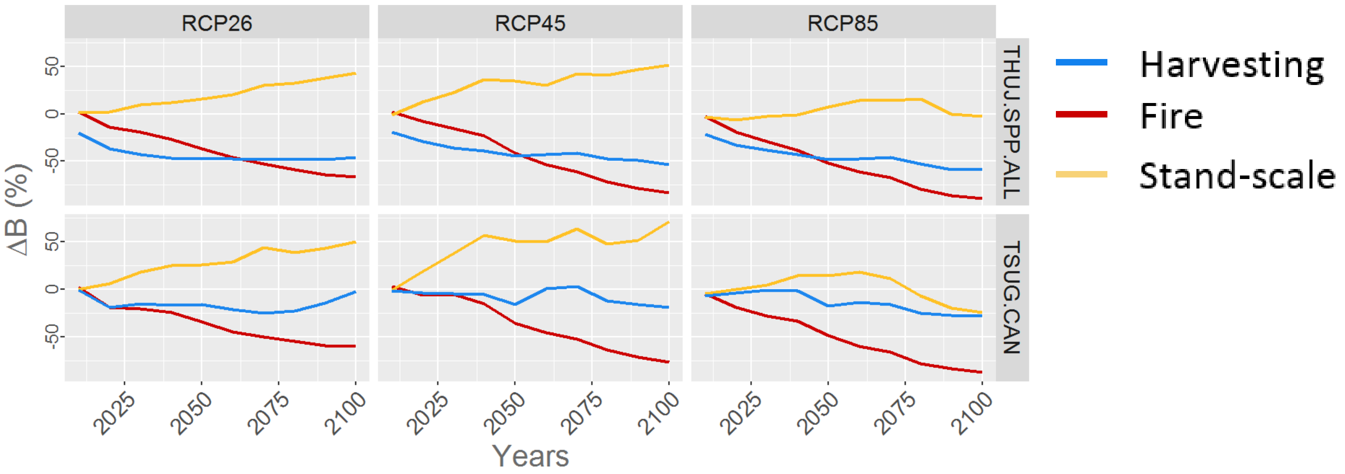

S5.1 Fig. Trends in species-specific mean biomass differences (ΔB) between the reduced and the full model for each of the mechanism of change considered (either climate-induced changes in fire, in stand-scale processes as well as harvest) under RCP 2.6, RCP 4.5 or RCP 8.5 forcing scenarios. See Table 1 for species abbreviations.
